# Manganese Detoxification by MntE Is Critical for Resistance to Oxidative Stress and Virulence of *Staphylococcus aureus*

**DOI:** 10.1128/mBio.02915-18

**Published:** 2019-02-26

**Authors:** Caroline M. Grunenwald, Jacob E. Choby, Lillian J. Juttukonda, William N. Beavers, Andy Weiss, Victor J. Torres, Eric P. Skaar

**Affiliations:** a Department of Pathology, Microbiology, and Immunology, Vanderbilt University Medical Center, Nashville, Tennessee, USA; b Vanderbilt Institute for Infection, Immunology, and Inflammation, Vanderbilt University Medical Center, Nashville, Tennessee, USA; c Graduate Program in Microbiology & Immunology, Vanderbilt University, Nashville, Tennessee, USA; d Department of Microbiology, New York University School of Medicine, New York, New York, USA; UT Southwestern Medical Center Dallas; University of Nebraska Medical Center; University of Maryland School of Pharmacy

**Keywords:** *Staphylococcus aureus*, iron metabolism, manganese, metal resistance, oxidative stress

## Abstract

Manganese (Mn) is an essential micronutrient critical for the pathogenesis of Staphylococcus aureus, a significant cause of human morbidity and mortality. Paradoxically, excess Mn is toxic; therefore, maintenance of intracellular Mn homeostasis is required for survival. Here we describe a Mn exporter in S. aureus, MntE, which is a member of the cation diffusion facilitator (CDF) protein family and conserved among Gram-positive pathogens. Upregulation of *mntE* transcription in response to excess Mn is dependent on the presence of MntR, a transcriptional repressor of the *mntABC* Mn uptake system. Inactivation of *mntE* or *mntR* leads to reduced growth in media supplemented with Mn, demonstrating MntE is required for detoxification of excess Mn. Inactivation of *mntE* results in elevated levels of intracellular Mn, but reduced intracellular iron (Fe) levels, supporting the hypothesis that MntE functions as a Mn efflux pump and Mn efflux influences Fe homeostasis. Strains inactivated for *mntE* are more sensitive to the oxidants NaOCl and paraquat, indicating Mn homeostasis is critical for resisting oxidative stress. Furthermore, *mntE* and *mntR* are required for full virulence of S. aureus during infection, suggesting S. aureus experiences Mn toxicity *in vivo*. Combined, these data support a model in which MntR controls Mn homeostasis by balancing transcriptional repression of *mntABC* and induction of *mntE*, both of which are critical for S. aureus pathogenesis. Thus, Mn efflux contributes to bacterial survival and virulence during infection, establishing MntE as a potential antimicrobial target and expanding our understanding of Mn homeostasis.

## Abstract (precis)

Manganese (Mn) is generally viewed as a critical nutrient that is beneficial to pathogenic bacteria due to its function as an enzymatic cofactor and its capability of acting as an antioxidant; yet paradoxically, high concentrations of this transition metal can be toxic. In this work, we demonstrate Staphylococcus aureus utilizes the cation diffusion facilitator (CDF) family protein MntE to alleviate Mn toxicity through efflux of excess Mn. Inactivation of *mntE* leads to a significant reduction in S. aureus resistance to oxidative stress and S. aureus*-*mediated mortality within a mouse model of systemic infection. These results highlight the importance of MntE-mediated Mn detoxification in intracellular Mn homeostasis, resistance to oxidative stress, and S. aureus virulence. Therefore, this establishes MntE as a potential target for development of anti-S. aureus therapeutics.

## INTRODUCTION

Metals, such as manganese (Mn), iron (Fe), and zinc (Zn), are essential nutrients for nearly all organisms and serve critical roles in cellular physiology as enzymatic cofactors and contributors to protein structure. Therefore, acquisition of metals and maintenance of optimal intracellular metal levels are paramount for pathogen survival and replication during infection. Recent evidence points to Mn as a key micronutrient that contributes to the pathogenesis of a variety of bacterial species (1, 2). This includes the Gram-positive bacterium Staphylococcus aureus, which is responsible for a diverse set of devastating diseases, including skin and soft tissue infections, osteomyelitis, septicemia, pneumonia, and endocarditis (1, 3).

The microbial requirement for nutrient metal is exploited by the vertebrate immune system, which produces metal-sequestering proteins that restrict divalent cations, including Mn, from bacterial pathogens in a process termed nutritional immunity (4, 5). This is best exemplified by the antimicrobial effects of the host protein calprotectin, which binds Mn, Zn, and other divalent cations and sequesters these metals at sites of infection (6–9). Calprotectin, a heterodimer of S100A8 and S100A9, constitutes the majority of the cytoplasmic proteome in neutrophils (10). However, the bioavailability of Mn within the host environment can vary widely. Host tissues inherently exhibit differences in Mn levels (11, 12). In healthy human hosts, Mn concentrations in blood and serum typically range from 20 to 200 nM (13). Liver and kidneys (20 to 50 nmol/g) are Mn rich compared to other organs, such as the brain, heart, and lungs, which have Mn concentrations of <20 nmol/g (13). Moreover, increases in dietary Mn significantly enhance the Mn content of the liver and heart and can impact the outcome of infection (11, 14). This suggests S. aureus must be capable of responding to altered Mn levels during infection.

As metals can neither be synthesized nor degraded, modulation of transport of Mn is an important process to maintain optimal intracellular Mn concentrations in S. aureus. S. aureus encodes two Mn acquisition systems: MntABC, an ATP-binding cassette Mn^2+^ permease, and the NRAMP homolog MntH (15). Expression of these systems in response to Mn limitation is controlled by the metalloregulatory protein and member of the DtxR family MntR (15). MntR functions as a transcriptional repressor of *mntABC*, preventing expression under conditions of sufficient Mn, and as an activator of *mntH*, upregulating expression under low-Mn conditions (15). Genetic inactivation of both Mn uptake systems results in reduced bacterial burdens in the liver and kidneys, and inactivation of *mntC* alone reduces virulence in an S. aureus sepsis model of infection (11, 16). Moreover, S. aureus contains genes (*sodA* and *sodM*) that encode two superoxide dismutase (SOD) enzymes that utilize Mn as a cofactor and are important contributors to the ability of S. aureus to survive neutrophil-mediated killing (9). SodA strictly requires Mn, whereas SodM utilizes either Mn or Fe, and MntABC-dependent Mn import significantly enhances SOD activity *in vitro* (16, 17). Combined, these results underscore the importance of Mn for S. aureus pathogenesis and resistance to oxidative stress.

Despite their importance to cellular physiology, an overabundance of essential transition metals, including Mn, is toxic and can lead to cell death. Although the mechanisms are not entirely defined, metal intoxication is predicted to be a result of mismetallation of noncognate enzymes and transcriptional regulators, inappropriate signaling to other metal transporters that are regulated allosterically, and/or disruption of redox cycling (18). Recent studies in Streptococcus spp. and Neisseria meningitidis suggest Mn detoxification through Mn efflux is an important component of bacterial pathogenesis. Streptococcus pyogenes is more susceptible to killing by human neutrophils *in vitro* when the gene encoding the Mn efflux pump MntE, a member of the cation diffusion facilitator (CDF) protein family, is deleted (19). Moreover, *mntE* mutants of S. pyogenes, Streptococcus pneumoniae, and Streptococcus suis demonstrate reduced pathogenesis in murine infection models (19–21). Mn efflux mediated by MntX is also important for N. meningitidis survival *in vivo* (22). Thus, sensing and tight regulation of intracellular Mn levels through both import and efflux are critical for bacterial survival within the host environment.

How S. aureus detoxifies excess Mn and what the consequences are of Mn intoxication have yet to be defined. It is estimated that approximately 30 to 45% of all known enzymes require a metal cofactor; therefore, intracellular metal levels must be tightly controlled to ensure proteins are populated with the proper metal species (18). S. aureus is capable of tolerating millimolar concentrations of Mn, implying S. aureus encodes a Mn detoxification system; however, the mechanism by which S. aureus senses and responds to Mn stress is unknown. Moreover, it is unclear in what environmental niches Mn detoxification is most important for S. aureus survival and pathogenesis.

In this study, we sought to identify the mechanisms by which S. aureus maintains intracellular Mn homeostasis to prevent metal intoxication and the contribution of Mn detoxification to staphylococcal pathogenesis. We describe a predicted CDF family protein (NWMN_2316) we have designated MntE, which functions to detoxify S. aureus of excess Mn and maintain optimal intracellular Mn concentrations. Under conditions of excess exogenous Mn, upregulation of *mntE* is the primary transcriptional response, and this is dependent on the transcriptional regulator MntR. Without MntE, S. aureus cells accumulate intracellular Mn, leading to a reduced tolerance to oxidative stress and a reduction in S. aureus*-*mediated lethality during systemic infection. Together, the findings in this report reveal Mn detoxification by MntE is critical for maintenance of optimal intracellular Mn levels and full virulence of S. aureus.

## RESULTS

### Transcriptional response of S. aureus to excess exogenous Mn.

**FIG 1:**
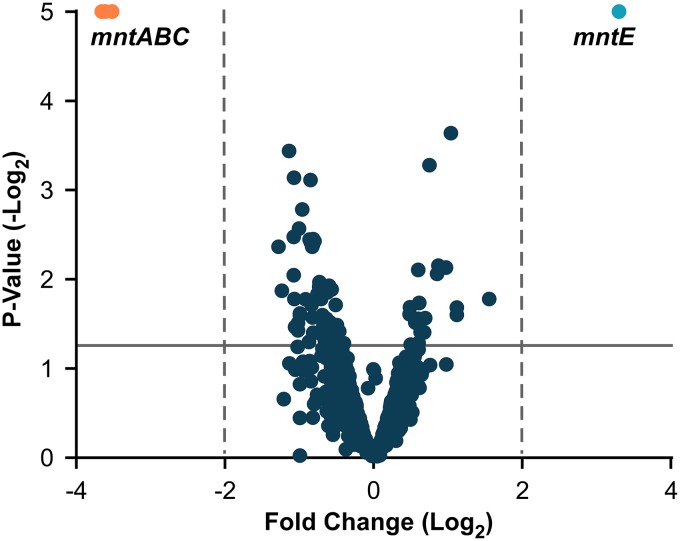
Exposure to 1 mM MnCl_2_ alters the S. aureus transcriptome. Shown are results from RNA sequencing analysis measuring fold change of transcript abundance for S. aureus WT cells treated with 1 mM MnCl_2_ compared to untreated controls. Genes with greater than 2-fold change (dashed lines) and a *P* value of >0.05 (solid line) are highlighted. The actual *P* values of *mntE* and *mntABC* are <1 × 10^−5^ (graphical cutoff).

To identify potential candidate genes involved in the detoxification of excess Mn and maintenance of intracellular Mn homeostasis, we investigated the transcriptional response of S. aureus cells exposed to 1 mM MnCl_2_ compared to untreated cells by using RNA sequencing (Fig. 1). Total transcript abundance was unchanged for the majority of S. aureus genes (see Table S1 in the supplemental material). A single transcript, *NWMN_2316*, which is predicted to encode a 288-amino-acid membrane protein in the CDF family, exhibited a significant 3.3-fold increase in response to Mn treatment. Other transcripts significantly changed include genes encoding the well-characterized Mn ABC transporter, MntABC, which exhibited a 3.5- to 3.6-fold decrease in transcript abundance. As *NWMN_2316* was the transcript with the highest positive fold change in response to Mn treatment and proteins of the CDF family function as cation efflux pumps, we hypothesized this gene is an important component of the Mn detoxification strategy of S. aureus. Based on these observations and the data described below, we renamed *NWMN_2316* as *mntE*.

10.1128/mBio.02915-18.6 TABLE S1 RNA sequencing results. Download Table S1, XLSX file, 0.7 MB. Copyright © 2019 Grunenwald et al. 2019 Grunenwald et al. This content is distributed under the terms of the Creative Commons Attribution 4.0 International license.

### MntE and its genomic context are conserved across staphylococci and other Gram-positive pathogens.

**FIG 2:**
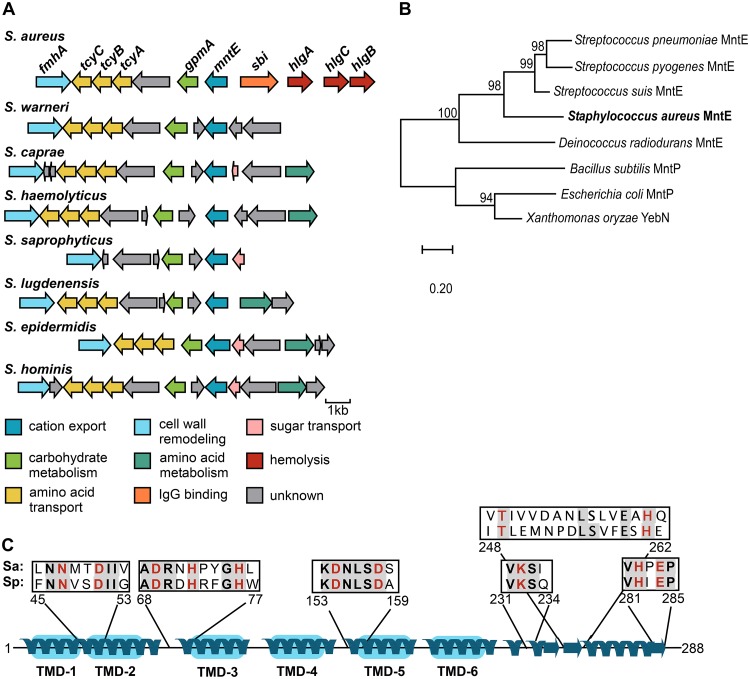
MntE is conserved among staphylococci and other Gram-positive organisms. (A) Conservation of *mntE* and its genomic context across staphylococci were assessed using the SEED Viewer database (pubseed.theseed.org). The genes shown share similar sequence and are color coded based on function. (B) Phylogenetic analysis of protein sequences from characterized bacterial Mn exporters and predicted S. aureus MntE. Amino acid sequences were aligned using Clustal Omega, and evolutionary history was inferred using the neighbor-joining method. The tree is drawn to scale, with bootstrap values (×1,000) shown above the nodes. (C) Predicted secondary structure of S. aureus MntE (288 amino acids) as determined by Phyre2 protein modeling portal. Predicted transmembrane domains (TMD) are shaded in blue. Boxes represent putative metal binding sites for S. aureus (Sa) MntE compared to Mn-binding sites previously identified in Streptococcus pneumoniae (Sp) MntE, where conserved residues are shaded and metal-binding residues are shown in red.

Orthologs of *mntE* were found in the genomes of all S. aureus strains and staphylococcus species available within the SEED Viewer database (Fig. 2A). Moreover, the genomic context was also conserved, as genes downstream of *mntE*, including *gpmA*, which encodes the Mn-requiring enzyme 2,3-bisphosphoglycerate-dependent phosphoglycerate mutase, were found in all species examined. Genes involved in cystine transport (*tcyABC*) and cell wall remodeling (*fmhA*) were also conserved. In contrast, upstream regions were not conserved across staphylococcal species.

Protein sequence alignment and phylogenetic analysis of S. aureus MntE with other characterized bacterial Mn efflux proteins revealed this protein is closely related to MntE of *Streptococcus* spp. (Fig. 2B), sharing 41 to 80% sequence identity. Consistent with other known bacterial efflux pumps of the CDF family, the predicted secondary structure of MntE contains six putative transmembrane domains (Fig. 2C). Furthermore, residues previously identified as critical for Mn binding in Streptococcus pneumoniae MntE are conserved in the S. aureus CDF protein (Fig. 2C; see Fig. S1 in the supplemental material) (23). Taken together, these data indicate MntE-mediated detoxification of Mn is a common homeostatic strategy among staphylococci and other bacterial species.

10.1128/mBio.02915-18.1 FIG S1 Multiple sequence alignment of MntE from Staphylococcus aureus and *Streptococcus* spp. MntE protein sequences were aligned by Clustal Omega. Conserved residues are shaded in gray, with asterisks denoting sequence conservation across all four species. Metal binding residues previously identified in S. pneumoniae (23) are highlighted in red. S. aureus MntE sequences share 41 to 80% sequence identity with those of *Streptococcus* spp. Download FIG S1, PDF file, 0.3 MB. Copyright © 2019 Grunenwald et al. 2019 Grunenwald et al. This content is distributed under the terms of the Creative Commons Attribution 4.0 International license.

### MntR is required for upregulation of *mntE* transcription in response to exogenous Mn.

**FIG 3:**
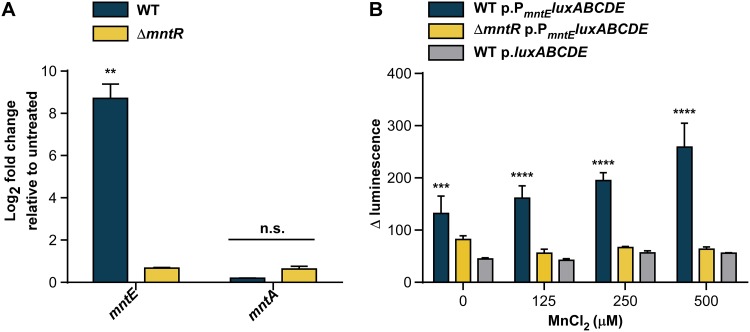
MntR upregulates *mntE* expression in response to exogenous Mn. (A) Relative transcript abundance of *mntE* and *mntA* mRNA isolated from mid-exponential growth of S. aureus strains grown in a rich medium untreated or treated with 1 mM MnCl_2_ was measured by qRT-PCR. Data are graphed as fold change relative to untreated cultures. The data are the average from a single experiment performed in biological triplicate with standard deviation shown. “n.s.” indicates no significance by two-way analysis of variance (ANOVA) with Sidak’s correction for multiple comparisons, comparing fold change of the transcripts among WT and Δ*mntR* strains. **, *P* < 0.01. (B) Change in bioluminescence was determined for S. aureus strains following incubation for 3 h in medium containing increasing amounts of MnCl_2_. The data are the averages from three independent experiments, each in biological triplicate with the standard error of the mean shown. Statistical significance was determined using a two-way ANOVA with Dunnett’s correction for multiple comparisons, comparing luminescence data for WT p.P*_mntE_luxABCDE* and Δ*mntR* p.P*_mntE_luxABCDE* against WT p.*luxABCDE* for each condition. ***, *P* < 0.001; ****, *P* < 0.0001.

Due to the role of Mn as an essential nutrient and the inherent toxicity of excess metals, we hypothesized *mntE* expression is tightly regulated. MntR is a transcriptional regulator of the DtxR family and the only staphylococcal regulator known to control the expression of genes encoding the Mn importers MntABC and MntH (15). Members of the DtxR family can function as bi-regulators, and MntR is known to control expression of Mn transport systems in other bacteria (24–26). Therefore, we hypothesized MntR may also play a role in the regulation of S. aureus MntE. Expression of *mntE* was significantly increased in wild-type (WT) S. aureus compared to a Δ*mntR* mutant in response to 1 mM MnCl_2_ compared to untreated controls, as measured by quantitative reverse transcriptase PCR (qRT-PCR) (Fig. 3A). Conversely, no change was detected in *mntA* transcription, which is upregulated under Mn-limited conditions, demonstrating 1 mM MnCl_2_ was sufficient to suppress the S. aureus Mn starvation response. To confirm these results, we created a bioluminescent reporter plasmid with 350 bp upstream of the predicted translational start site of *mntE* driving the expression of a luciferase operon, and measured *mntE* reporter activity. Luminescence significantly increased in a dose-dependent response to increasing concentrations of MnCl_2_ in WT strains, but not in the Δ*mntR* strain, compared to promoterless controls (Fig. 3B). Combined, these data indicate S. aureus senses increasing concentrations of Mn and suggest that MntR contributes to the transcriptional activation of *mntE* expression to alleviate Mn toxicity.

### *S. aureus* requires *mntE* for Mn detoxification.

**FIG 4:**
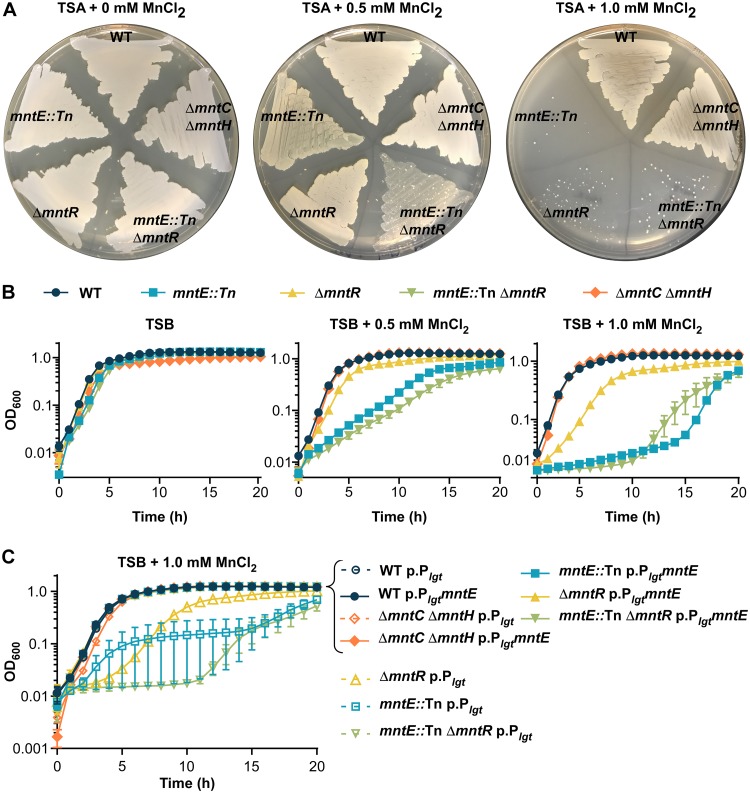
S. aureus requires *mntE* for growth in the presence of excess Mn. (A) S. aureus strains streaked onto TSA plates supplemented with 0, 0.5, and 1.0 mM MnCl_2_. (B) Growth as measured by OD_600_ over time for S. aureus strains in TSB medium supplemented with 0, 0.5, or 1.0 mM MnCl_2_. The data are the average of the means from three independent experiments each in biological triplicate with the standard error of the mean shown. (C) Growth as measured by OD_600_ over time for S. aureus strains containing either an empty plasmid (pOS1 Plgt [p.P*_lgt_*]) or a plasmid containing constitutively expressed *mntE* (pOS1 P*_lgt_mntE* [p.P*_lgt_mntE*]) in TSB supplemented with 1 mM MnCl_2_. The data are the average of the means from three independent experiments, each in biological triplicate with the standard error of the mean shown.

Metal toxicity that results in growth arrest is thought to be a result of mismetallation of noncognate enzymes, the dysregulation of metal-dependent signaling, and/or the disruption of redox cycling (18). Therefore, we hypothesized inactivation of *mntE* would disrupt Mn detoxification and lead to increased sensitivity to exogenous Mn. Inactivation of *mntE* resulted in substantially decreased growth on solid media and in liquid cultures when supplemented with 0.5 or 1 mM MnCl_2_ (Fig. 4A and B). Due to our observation that MntR is required for activation of *mntE* transcription in response to excess Mn and MntR represses *mntABC* under sufficient-Mn conditions, we predicted inactivation of *mntR* would also result in increased sensitivity to Mn. Δ*mntR* and *mntE*::Tn Δ*mntR* mutants exhibited decreased growth on solid media and liquid cultures supplemented with MnCl_2_, with the *mntE*::Tn Δ*mntR* mutant experiencing the most attenuated growth in liquid cultures (Fig. 4A and B). No differences in growth were observed for strains with *mntC* and *mntH* inactivated compared to WT S. aureus, demonstrating the growth conditions contained sufficient Mn levels. Constitutive expression of *mntE* in *trans* completely restored growth of both Δ*mntR* and *mntE*::Tn mutants in the presence of 1 mM MnCl_2_, further supporting the hypothesis that *mntE* is required for Mn detoxification and MntR is required for transcriptional activation of *mntE* (Fig. 4C).

**FIG 5:**
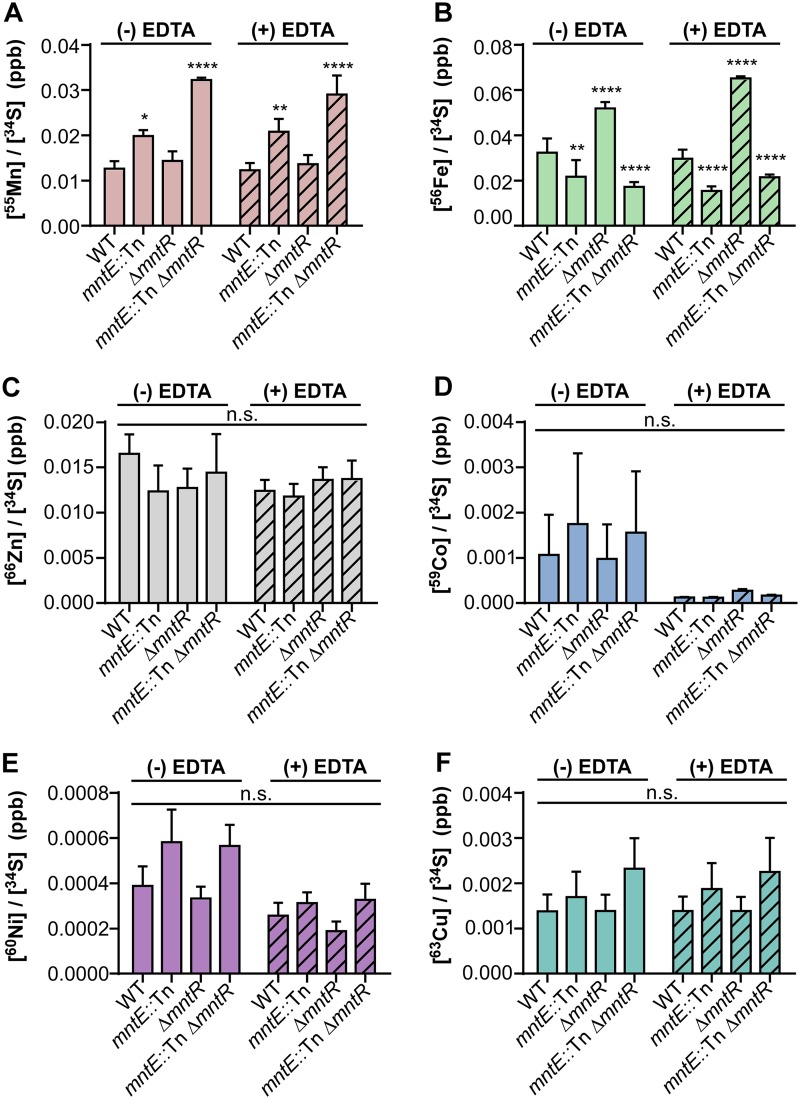
Inactivation of *mntE* leads to accumulation of intracellular Mn and a reduction in intracellular Fe levels. Concentrations of intracellular Mn (A), Fe (B), Zn (C), Co (D), Ni (E), and Cu (F) were measured using inductively coupled plasma mass spectrometry and normalized to intracellular sulfur for S. aureus strains grown under metal-replete (TSB) and metal-deplete (TSB plus 100 mM EDTA) conditions. The data are the average of the means from three independent experiments, each in biological triplicate with error bars representing standard error. *, *P* < 0.05, **, *P* < 0.01, and ****, *P* < 0.0001, calculated by two-way ANOVA with Dunnett’s correction for multiple comparisons, comparing metal levels for each strain with WT S. aureus. n.s., not significant.

Many CDF proteins are capable of removing multiple cation species from the cytoplasm, including the well-characterized YiiP of Escherichia coli (Zn/Fe) and CzcD of Bacillus subtilis (Zn/Co/Cd) (27–29). To investigate the specificity of S. aureus MntE, we measured intracellular metal levels of S. aureus strains grown in rich media following an overnight outgrowth in rich media (metal replete) or media treated with EDTA (metal deplete) using inductively coupled plasma mass spectrometry (ICP-MS) (Fig. 5). Under both metal-replete and metal-deplete conditions, strains inactivated for *mntE* accumulated significantly higher concentrations of intracellular Mn compared to WT S. aureus; however, there was no difference in intracellular Mn levels between the Δ*mntR* and WT strains. This phenotype can be complemented when *mntE* is constitutively expressed in *trans* (see Fig. S2A in the supplemental material). Interestingly, inactivation of *mntR* led to significantly higher levels of intracellular Fe, which can be suppressed by inactivation of *mntE.* Significantly lower intracellular Fe levels were observed in *mntE*::Tn and Δ*mntR mntE*::Tn strains compared to WT S. aureus, implying an inverse relationship between intracellular Mn and Fe levels (Fig. 5A and B). When *mntE* is constitutively expressed in *trans*, Fe levels return to WT levels in the *mntE*::Tn strain and the *mntE*::Tn Δ*mntR* phenotype mimics that of the Δ*mntR* strain (Fig. S2B). No differences were observed in the intracellular concentration of other metals, including Zn, copper (Cu), cobalt (Co), and nickel (Ni) among strains, suggesting MntE efflux is preferential to Mn, and Mn efflux affects intracellular Fe levels (Fig. 5C to F).

10.1128/mBio.02915-18.2 FIG S2 Intracellular metal concentrations are restored to WT S. aureus levels when *mntE* is constitutively expressed. Concentrations of intracellular Mn and Fe were measured using inductively coupled plasma mass spectrometry and normalized to intracellular sulfur for S. aureus strains grown in TSB. The data are the mean from three independent experiments, each in biological triplicate with error bars representing standard error. n.s., not significant, *, *P* < 0.05, ***, *P* < 0.001, and ****, *P* < 0.0001, calculated by two-way ANOVA with Dunnett’s correction for multiple comparisons, comparing metal levels for each strain with WT S. aureus containing either empty vector (p.P*_lgt_*) or *mntE* complement (p.P*_lgt_mntE*). Download FIG S2, PDF file, 0.06 MB. Copyright © 2019 Grunenwald et al. 2019 Grunenwald et al. This content is distributed under the terms of the Creative Commons Attribution 4.0 International license.

### Mn efflux is required for resistance to oxidative stress.

**FIG 6:**
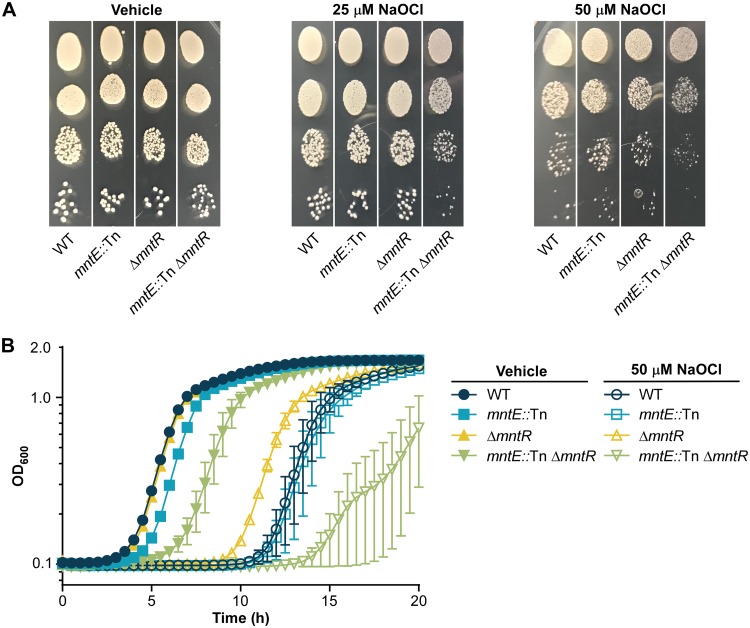
Inactivation of *mntE* leads to increased sensitivity to NaOCl-mediated oxidative stress. (A) Colony morphology of S. aureus strains following a 30-min exposure to PBS containing 25 or 50 mM NaOCl was assessed by serially diluting cells and plating onto TSA. Images of individual plates containing a single S. aureus strain were combined for each treatment to facilitate direct phenotypic comparisons within treatment groups. (B) Growth as measured by OD_600_ over time in TSB following a 30-min exposure to PBS containing 0 or 50 mM NaOCl. The data are the average of the means from two independent experiments, each in biological triplicate with the standard error of the mean shown.

Despite the ability of Mn to serve as an antioxidant, excess Mn is inherently toxic, possibly due to increased oxidative stress. Thus, we hypothesized the increase in intracellular Mn by direct loss of *mntE*, or by reducing *mntE* expression through inactivation of *mntR*, would alter the ability of S. aureus to resist oxidative stress. To test this hypothesis, we performed an oxidative pulse experiment in which S. aureus strains were exposed to either the oxidative stressor sodium hypochlorite (NaOCl) or phosphate-buffered saline (PBS) for 30 min, serially diluted, and then plated onto tryptic soy agar (TSA) (Fig. 6A). NaOCl produces hypochlorous acid (HOCl), an oxidant that is produced within the phagolysosome of macrophages and neutrophils as part of the respiratory burst used to kill invading pathogens. Inactivation of both *mntE* and *mntR* led to alterations in colony phenotype compared to WT or single mutant strains when cells were statically exposed to 25 μM NaOCl, where colonies formed by the double mutants were smaller. This phenotype was enhanced in the *mntE*::Tn Δ*mntR* mutant following treatment with 50 μM NaOCl. No differences in colony morphology were observed between strains treated with PBS. Additionally, we assessed the ability of S. aureus strains to grow in the presence of NaOCl (Fig. 6B). Inactivation of both *mntE* and *mntR* resulted in a severe growth defect compared to the WT, *mntE*::Tn, or Δ*mntR* strain following a 50 μM NaOCl treatment. Therefore, we concluded maintenance of Mn homeostasis coordinated by *mntE* and *mntR* contributes to the resistance of S. aureus to NaOCl-induced oxidative stress.

### Mn efflux contributes to *S. aureus*-mediated lethality and pathogenesis.

**FIG 7:**
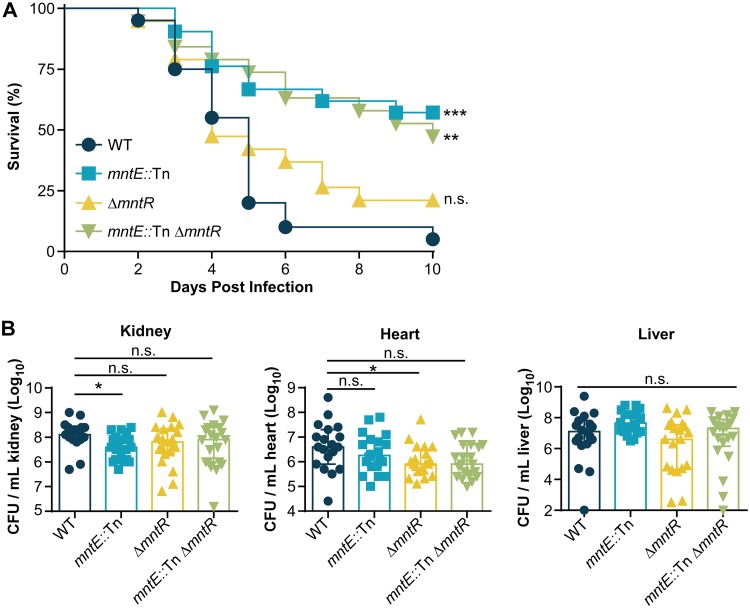
*mntE* contributes to S. aureus-mediated lethality and pathogenesis during systemic infection. (A) Survival of mice infected with 3 × 10^7^ WT and mutant bacteria was monitored over time. WT, *n* = 20; *mntE*::Tn mutant, *n* = 21; Δ*mntR* mutant, *n* = 19; *mntE*::Tn Δ*mntR* mutant, *n* = 19. n.s., not significant, **, *P* < 0.01, and ***, *P* < 0.001, calculated by log-rank test comparing percentage of survival of mice infected with mutant strains against mice infected with WT S. aureus. (B) Colony-forming units (CFU) present in organ homogenates 4 days postinfection from mice infected retro-orbitally with 1 × 10^7^ bacteria. WT, *n* = 19; *mntE*::Tn mutant, *n* = 20; Δ*mntR* mutant, *n* = 21; *mntE*::Tn Δ*mntR* mutant, *n* = 21. The limit of detection is 1 × 10^2^. Error bars depict median and interquartile range. n.s., not significant. *, *P* < 0.05, calculated by Kruskal-Wallis test with Dunn’s correction for multiple comparisons (kidney, heart, and liver) comparing mutant strains against the WT.

During infection, Mn is required for S. aureus survival and proliferation within the host; however, it is unclear what role Mn efflux serves in S. aureus*-*mediated pathogenesis and virulence. To investigate the contribution of *mntE* to S. aureus virulence, 6-week-old female BALB/cJ mice were retro-orbitally infected with S. aureus and monitored for 10 days (Fig. 7A). The mortality rate following systemic infection was significantly reduced when mice were infected with *mntE*::Tn or *mntE*::Tn Δ*mntR* strains, but not the Δ*mntR* strain, compared to WT-infected animals (Fig. 7A). To assess the contribution of Mn homeostasis to bacterial dissemination and survival, 6-week-old female BALB/cJ mice were retro-orbitally infected with S. aureus and monitored for 4 days postinfection, and bacteria were enumerated from the heart, kidneys, and liver. Despite the difference in mortality rate, few notable differences in organ bacterial burdens were observed among mice infected with S. aureus WT and mutant strains (Fig. 7B). There were significantly fewer *mntE*::Tn mutant bacteria recovered in the kidneys and significantly fewer Δ*mntR* bacteria recovered from the hearts; however, no other significant differences were observed among strains in those tissues. No differences were observed in the number of bacteria recovered from the livers of mice infected with the various strains. Moreover, increasing the length of the infection to 10 days did not reveal any significant differences in bacterial burdens between S. aureus strains in murine hearts, livers, or kidneys (see Fig. S3 in the supplemental material).

10.1128/mBio.02915-18.3 FIG S3 S. aureus bacterial burdens following 10 days of infection. Shown are colony-forming units (CFU) present in organ homogenates 10 days postinfection from mice infected retro-orbitally with 1 × 10^7^ bacteria. WT, *n* = 8; *mntE*::Tn mutant, *n* = 7; Δ*mntR* mutant, *n* = 8; *mntE*::Tn Δ*mntR* mutant, *n* = 7. The limit of detection is 1 × 10^2^. Error bars depict median and interquartile range. n.s., not significant where α = 0.05, calculated by Kruskal-Wallis test with Dunn’s correction for multiple comparisons comparing mutant strains against the WT. Download FIG S3, PDF file, 0.07 MB. Copyright © 2019 Grunenwald et al. 2019 Grunenwald et al. This content is distributed under the terms of the Creative Commons Attribution 4.0 International license.

### Inactivation of *mntE* reduces fitness of *S. aureus* strains *in vivo*.

**FIG 8:**
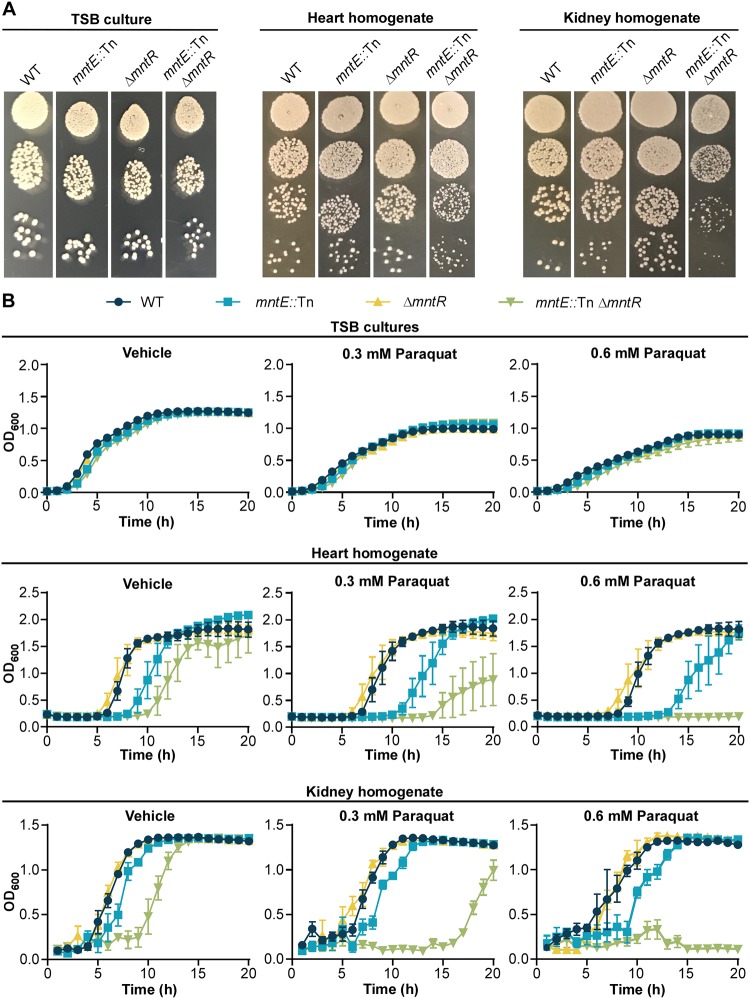
S. aureus *mntE* mutants isolated from murine organs exhibit reduced fitness. (A) Colony morphology of S. aureus strains isolated from murine hearts and kidneys compared to strains grown in a rich medium. Eight-hour liquid cultures of S. aureus strains grown in TSB were serially diluted in PBS and plated onto TSA. Organs from mice retro-orbitally infected with 1 × 10^7^ CFU were harvested 4 days postinfection and homogenized in PBS, and serial dilutions were plated onto TSA medium. Images were taken approximately 15 h following incubation at 37°C. (B) Growth as measured by OD_600_ over time for murine heart and kidney homogenates in TSB medium treated with 0, 0.3, or 0.6 mM paraquat. Organs from mice retro-orbitally infected with 1 × 10^7^ CFU were harvested 4 days postinfection, homogenized in PBS, and added 1:100 to the media. The data are the representative of one of two independent experiments, each performed in biological triplicate with the standard deviation of the mean shown.

The loss of *mntE* results in reduced resistance to NaOCl-mediated oxidative stress; therefore we hypothesized the reduction in mortality observed in mice infected with the *mntE*::Tn mutant S. aureus strains could be a result of increased susceptibility to oxidative damage. *mntE*::Tn and *mntE*::Tn Δ*mntR* strains from heart and kidney homogenates plated onto TSA exhibited a similar irregular colony phenotype (Fig. 8A), as was observed when strains were treated with NaOCl (Fig. 6A). However, this phenotype is reversible as *mntE*::Tn and *mntE*::Tn Δ*mntR* colonies revert back to the WT colony morphology following additional incubation or a single *in vitro* passage (see Fig. S4 in the supplemental material). Thus, we predicted that strains inactivated for *mntE* were weakened by *in vivo* growth and exposure to an oxidative stressor would further damage these strains. Unlike NaOCl, each strain exhibits equal sensitivity to the superoxide-producing agent paraquat when grown *in vitro* (Fig. 8A). Therefore, we hypothesized decreased fitness due to *mntE* inactivation and subsequent damage during *in vivo* passage would result in increased sensitivity to paraquat compared to the WT strain. To test this, we exposed bacteria from murine kidney and heart homogenates that were infected with WT, *mntE*::Tn, Δ*mntR*, and *mntE*::Tn Δ*mntR* strains to paraquat (Fig. 8B). All strains cultured directly from organ homogenates demonstrated similar growth kinetics in vehicle-treated media. Conversely, following *in vivo* passage, strains inactivated for *mntE* or both *mntE* and *mntR* displayed extended lag phases in vehicle-treated media alone, despite equal numbers of CFU serving as the experimental inoculum (see Fig. S5 in the supplemental material). Moreover, this phenotype was dramatically enhanced by the addition of paraquat. Taken together, these data suggest MntE is critical for fitness of S. aureus *in vivo*, and that S. aureus must efflux Mn *in vivo* to resist oxidative damage.

10.1128/mBio.02915-18.4 FIG S4 Strains with inactivated *mntE* revert to WT colony morphology following extended incubation or *in vitro* passage. Shown is the colony morphology of S. aureus strains isolated from murine hearts and kidneys. Organs from mice retro-orbitally infected with 1 × 10^7^ CFUs were harvested 10 days postinfection and homogenized in PBS, serial dilutions were plated onto TSA medium, and plates were incubated at 37°C. Images were taken after approximately 15 h and 48 h of incubation. Single colonies were also selected from the plates following 15 h of incubation and inoculated into TSB. After 15 h of growth, S. aureus liquid cultures were serially diluted in PBS and plated onto TSA. Download FIG S4, PDF file, 30.1 MB. Copyright © 2019 Grunenwald et al. 2019 Grunenwald et al. This content is distributed under the terms of the Creative Commons Attribution 4.0 International license.

10.1128/mBio.02915-18.5 FIG S5 S. aureus bacterial burdens of organ homogenates used in growth experiments to assess in vivo bacterial fitness. Shown are colony-forming units (CFU) present in organ homogenates 4 days postinfection from mice infected retro-orbitally with 1 × 10^7^ bacteria. Homogenates were used to inoculate growth curves in Fig. 8. WT, *n* = 6; *mntE*::Tn mutant, *n* = 6; Δ*mntR* mutant, *n* = 6; *mntE*::Tn Δ*mntR* mutant, *n* = 6. The limit of detection is 1 × 10^2^. Error bars depict the median and interquartile range. n.s., not significant where α = 0.05, calculated by Kruskal-Wallis test with Dunn’s correction for multiple comparisons comparing mutant strains against the WT. Download FIG S5, PDF file, 0.05 MB. Copyright © 2019 Grunenwald et al. 2019 Grunenwald et al. This content is distributed under the terms of the Creative Commons Attribution 4.0 International license.

## DISCUSSION

As a required nutrient for life, Mn acquisition is critical for bacterial survival within the vertebrate host. Accordingly, many species have evolved high-affinity transporters to enhance acquisition of Mn and promote survival within the host environment. S. aureus relies on the ABC-type MntABC and NRAMP MntH transporters to import Mn, which enable the pathogen to counteract the antimicrobial effects of calprotectin-mediated nutritional immunity (11). Insufficient intracellular Mn can have wide-ranging consequences, affecting Mn-dependent enzymes involved in transcription, metabolism, and defense against oxidative stress (9, 15, 16, 30–32). In this regard, it is unsurprising that inactivation of these transporters reduces S. aureus organ burdens during a murine model of infection (11). However, the inherent toxicity of excess Mn coupled with previous work establishing the contribution of Mn efflux in streptococcal pathogenesis and the number of putative Mn-specific efflux proteins that were recently inferred using comparative bioinformatics in other bacterial pathogens underscores the importance of Mn detoxification to bacterial pathogens (19–21, 33).

In this report, we identify a CDF protein we have named MntE that is required for S. aureus detoxification of Mn and is conserved among staphylococcal species (Fig. 2). MntE functions to alleviate excess Mn from the cytoplasm, and inactivation of *mntE* renders S. aureus susceptible to Mn toxicity and subsequent growth arrest (Fig. 4 and 5). Upregulation of *mntE* and downregulation of *mntABC* expression are the predominant transcriptional response to the addition of exogenous 1 mM MnCl_2_, demonstrating MntE is the major Mn detoxification system under these conditions (Fig. 1 and 2). The S. aureus DtxR protein MntR bidirectionally regulates the import and export of Mn by repressing *mntABC* and activating *mntE* expression in response to endogenous Mn (Fig. 2) (15). This implies S. aureus intracellular Mn levels are tightly controlled by modulating Mn import and export in response to environmental conditions. However, a strain inactivated for *mntR* does not accumulate intracellular Mn to the same extent as an *mntE*::Tn mutant (Fig. 5A). This could be explained by the basal transcription of *mntE* we observed in WT S. aureus grown in tryptic soy broth (TSB) (Table S1), but it is also possible *mntE* is regulated by additional mechanisms.

During infection, oxidative stress is an important challenge S. aureus must overcome to survive. The oxidants generated by neutrophils react with macromolecules to damage cellular lipids, proteins, and DNA. Mn-cofactored superoxide dismutases are critical for resisting phagocyte-produced superoxide, and low-molecular-weight Mn complexes have antioxidant properties that may also serve to detoxify reactive oxygen species (9, 34). The results reported here indicate elevated intracellular Mn following inactivation of *mntE* also leads to enhanced susceptibility to oxidative stressors in S. aureus (Fig. 6 and 8). Concordant with these results are observations in *Streptococcus* spp., where loss of MntE function results in reduced bacterial growth in the presence of hydrogen peroxide or the oxidizing sulfhydryl reagent diamide, for S. pyogenes and S. suis, respectively (19, 21). Therefore, disruption of intracellular Mn homeostasis either through excess or depletion can reduce the fitness of bacterial pathogens during infection.

Interestingly, the degree of difference in susceptibility to oxidative stress between S. aureus strains was dependent on the chemical oxidant applied and growth conditions (Fig. 6 and 8). When grown in a rich medium, strains inactivated for *mntE* and *mntR* exhibited reduced growth kinetics compared to WT strains when treated with NaOCl, yet no differences were observed between strains treated with paraquat. Paraquat generates superoxide, which, while not a potent oxidant itself, is a precursor to other damaging molecules, such as H_2_O_2_, and hydroxyl radicals (35, 36). NaOCl forms HOCl, which can react with amino acids to produce oxidizing chloramines that can serve as oxidants themselves and damage other cellular macromolecules (37). One possible explanation could be differences in which macromolecules are targeted and the degree of cellular damage that is produced by the presence of each oxidant; however, the mechanisms by which H_2_O_2_, HOCl, and other oxidants induce bacterial killing are still not entirely agreed upon (37). Moreover, S. aureus strains grown *in vivo* would experience nutrient limitation and more extensive oxidative damage due to host immune defenses, compared to those grown in a rich medium. Thus, the more dramatic fitness defect observed in response to paraquat treatment in strains with inactivated *mntE* and *mntR* is likely due to a synergistic effect of combined stressors (Fig. 8). Together, these results emphasize the impact of intracellular manganese homeostasis on S. aureus fitness and stress responses.

One possible mechanism contributing to the fitness defect observed in S. aureus strains inactivated for *mntE* could be the disruption of the intracellular Mn/Fe ratio. These data demonstrate the function of MntE significantly impacts the cytosolic concentrations of Fe, where the *mntE*::Tn and Δ*mntR mntE*::Tn strains had reduced Fe levels compared to the WT and Δ*mntR* strains (Fig. 5B). Curiously, the Δ*mntR* mutant contained significantly higher quantities of intracellular Fe, which could be the result of MntABC-mediated transport of Fe. However, previous work in Bacillus anthracis suggests MntC is unable to bind Fe, and thus, direct evidence that MntABC transports Fe is lacking (38). Displacement of Fe by Mn in Fe-requiring proteins would liberate free Fe into the cytoplasm, promoting oxidative damage incurred by Fenton chemistry. Furthermore, Mn has been shown to occupy Fe binding sites of the ferric uptake regulator (Fur) in B. subtilis, aberrantly repressing the expression of Fe uptake systems (39, 40). In appropriate repression of Fe acquisition systems due to increased intracellular Mn could explain the differences in cytosolic Fe levels observed between WT and *mntE-*inactivated S. aureus strains (Fig. 5B). Mn concentrations also influence the activity of the Fur family transcriptional regulator PerR, which controls the expression of genes involved in detoxifying reactive oxygen species, as well as iron homeostasis (39, 41–44). It is possible the increased intracellular Mn in an *mntE*::Tn mutant binds to PerR, repressing expression of the PerR regulon, including the catalase gene *katA* and *fur* (15, 45). This would limit the ability of S. aureus to mount an appropriate response to oxidative stress as well as disrupt *fur-*dependent expression of iron acquisition systems. Additional investigation of the connection between intracellular Mn and Fe levels is needed to uncover the impact of Mn toxicity on metal-related systems.

Although our data provide evidence that S. aureus experiences Mn intoxication *in vivo*, when and where this occurs during the course of an infection remains elusive. Mn levels among host populations can vary based on exposure and diet (14, 46). Moreover, Mn levels are not uniform across individual host tissues, and these levels can significantly impact infection outcomes (11, 14). Mice fed diets high in Mn accumulate Mn within the heart, leading to enhanced colonization of that tissue and increased mortality (14). S. aureus is capable of infecting nearly all body sites, suggesting that S. aureus is adapted to survive under a wide range of environmental Mn concentrations. Alternatively, Mn intoxication could be a consequence of the activity of the high-affinity importers MntABC and MntH. Upregulation of these systems in response to calprotectin-mediated Mn limitation could lead to a sudden influx of Mn. Therefore, it is possible MntE is required to buffer the abrupt change in the intracellular Mn concentration to prevent Mn toxicity, in a “binge and purge”-type model. Host-imposed Fe limitation could be another scenario, where Mn efflux is required to ensure a proper Mn/Fe ratio when Fe is restricted. Another possibility by which S. aureus experiences Mn intoxication could be within the phagolysosome. Recent data demonstrate metal intoxication is an antimicrobial strategy employed by phagocytes (47, 48). Upon engulfment, Cu and Zn accumulate within the phagocytic vacuole to induce intoxication. However, evidence of exogenous Mn intoxication as a killing strategy has not been uncovered. Instead, it is generally accepted that the phagolysosome is a Mn-poor environment due to the activity of host NRAMP1 transporters that deplete Mn from the phagocytic vacuole (49–51). Additional work to uncover the environmental niches in which MntE is essential is needed.

Data reported in this article suggest that MntE is a critical contributor to S. aureus virulence. Notably, mortality was significantly reduced for mice infected with *mntE*::Tn mutants (Fig. 7A). One possible mechanism contributing to the observed virulence defect may involve oxidative damage. As mentioned above, MntE-dependent removal of Mn from the cytoplasm alters S. aureus resistance to oxidative stress, and this phenotype is further exaggerated in mutants with inactivated *mntE* isolated from murine organs (Fig. 8). The small-colony morphology observed in these mutants is consistent with colony phenotypes previously reported as a response to oxidative damage, suggesting that *mntE*::Tn mutants experience enhanced oxidative stress *in vivo* (52). Increased oxidative damage would lead to reduced fitness and a redirection of resources to essential pathways instead of the production of potent, but energy-costly virulence factors. Additionally, as proposed above, mismetallation of Fur with Mn or a decrease in *fur* transcription by way of PerR-mediated repression could also alter virulence factor function. Previous work has determined that Fur positively regulates the Sae and Agr regulatory networks under low-iron conditions (53). Therefore, disruption of Fur-Fe sensing would have a considerable impact downstream on the expression of genes involved in immune evasion, biofilm formation, and oxidative stress response (53–57). This is further evidenced by the absence of several cytolysins and hemolysins under low-iron conditions and the significant virulence defect in a murine pneumonia model observed when *fur* is deleted (58). Additional work examining the impact of Mn homeostasis on virulence factor production would provide clarity into the mechanism by which Mn detoxification contributes to S. aureus-mediated lethality.

## MATERIALS AND METHODS

### Bacterial strains and growth conditions.

Bacterial strains (Table 1), plasmids (see Table S2 in the supplemental material), and primers (Table S2) are listed in the specified tables. Unless noted, S. aureus strains were routinely cultured on tryptic soy agar (TSA) or tryptic soy broth (TSB) supplemented with 10 μg/ml erythromycin or 10 μg/ml chloramphenicol when needed. When used, MnCl_2_ was prepared as a 1 M stock in sterile water and an equal volume of water was added for all conditions. E. coli strains were grown on lysogeny broth (LB) or LB agar (LBA), supplemented with 50 μg/ml carbenicillin when necessary. All growth in liquid medium occurred in an Innova 44 incubator shaking at 180 rpm, unless otherwise noted. Fifteen-milliliter round-bottom polypropylene tubes with aeration lids at a 45° angle were used for all standard cultures of 5 ml. Unless otherwise noted, all chemicals were from Sigma. All molecular biology reagents were from New England Biolabs and were used according to the manufacturer’s instructions. Phusion 2× Hi-fidelity master mix was used for all PCRs. For construction of plasmids, all constructs were confirmed by Sanger sequencing (GeneWiz). All plasmids were transformed by electroporation from E. coli into the S. aureus cloning intermediate strain RN4220. Plasmid extractions and purifications were performed using a GeneJet plasmid miniprep kit (Thermo Fisher). Following isolation from RN4220, plasmids were transduced into final S. aureus strains with bacteriophage ϕ85. Briefly, recipient strains were grown for 15 h in TSB at 37°C with 180 rpm of shaking. One hundred microliters of bacteriophage ϕ85 isolated from RN4220 donor strains was combined with 1 ml of a 15-h recipient strain culture and 1 ml of phage buffer containing 1 mM MgSO_4_, 4 mM CaCl_2_, 50 mM Tris-HCl_2_ (pH 7.8), 0.59% NaCl, and 0.1% gelatin. Bacteriophage reaction mixtures were incubated for 20 min in a 37°C water bath, followed by the addition of 2 ml of 1% sodium citrate. Bacterial cells were pelleted, resuspended in 2 ml TSB containing 0.5% sodium citrate, and incubated in a 37°C water bath for 1 h. Bacterial cells were centrifuged, and the cell pellets were resuspended in TSB supplemented with 0.5% sodium citrate and then plated onto TSA plates containing 0.5% sodium citrate and 10 μg/ml chloramphenicol to select for transduction of the plasmid.

**TABLE 1:** S. aureus strains used in this study

Strain	Genotype	Description	Source or reference
Newman	WT	Wild-type, methicillin-sensitive clinical isolate	69
JE2	*mntE*::Tn	*mntE*::Tn(NE418)	BEI (60)
Newman	*mntE*::Tn	*mntE*::Tn(NE418) transduced into Newman WT	This study
Newman	Δ*mntR*	In-frame unmarked deletion of *mntR* generated by allelic exchange	This study
Newman	*mntE*::Tn Δ*mntR*	*mntE*::Tn(NE418) transduced into Δ*mntR*	This study
Newman	Δ*mntC* Δ*mntH*	In-frame unmarked deletion of *mntC* and *mntH* generated by allelic exchange	11
RN4220		Restriction-deficient cloning-intermediate strain	70

10.1128/mBio.02915-18.7 TABLE S2 Plasmids and primers used in this study. Download Table S2, DOCX file, 0.01 MB. Copyright © 2019 Grunenwald et al. 2019 Grunenwald et al. This content is distributed under the terms of the Creative Commons Attribution 4.0 International license.

### Transduction of transposon library alleles.

Transduction of the *mntE*::Tn(NE418) transposon allele into Newman WT and Δ*mntR* strains was performed using bacteriophage ϕ85 as described previously (59, 60). Alleles were confirmed by colony PCR with primers CMG-176 and CMG-177, as well as by Sanger sequencing.

### Conservation of genomic context of *mntE*.

The genomic context of *mntE* among staphylococci was assessed using the SEED Viewer database (pubseed.theseed.org [accessed January 2018]) (61). A 16-kb region was compared across genomes within the SEED database to identify genes whose sequence and relative position are conserved. Nineteen species from the *Staphylococcus* genus were identified, and based on conserved patterns of gene ontology, the following 8 species with the most similarity were chosen to be represented graphically: S. aureus strain Newman, Staphylococcus warneri strain L376, Staphylococcus caprae strain C87, Staphylococcus haemolyticus strain R1P1, Staphylococcus saprophyticus strain ATCC 15305, Staphylococcus lugdunensis strain M23590, Staphylococcus epidermidis strain NIHLM037, and Staphylococcus hominis strain ZBW5.

### MntE multiple sequence alignment and secondary structure prediction.

The MntE multiple sequence alignment was performed using Clustal Omega (http://www.clustal.org/omega/ [accessed January 2018]) (62) using Staphylococcus aureus strain Newman (NCBI accession no. BAF68588.1), Streptococcus suis (NCBI accession no. WP_012775167.1), Streptococcus pneumoniae (NCBI accession no. WP_000813921.1), Streptococcus pyogenes (NCBI accession no. WP_010922370.1), Escherichia coli strain K-12 (NCBI accession no. NP_416335.4), Bacillus subtilis (NCBI accession no. WP_014481191.1), Xanthomonas oryzae (NCBI accession no. WP_011407353.1), and Deinococcus radiodurans (NCBI accession no. AAF10804.1). Phylogenetic relationships were inferred by the neighbor-joining method (bootstrap replicates ×1,000) using MEGA7 software (63). Phylogenetic trees were edited using FigTree v1.4.3 (http://tree.bio.ed.ac.uk/software/figtree). MntE secondary structure and transmembrane domain topology were predicted using Phyre2 (http://www.sbg.bio.ic.ac.uk/phyre2 [accessed January 2018]) as described previously (64). The S. aureus MntE model was confirmed using PSPIRED v3.3 and MEMSTAT-SVM (http://bioinf.cs.ucl.ac.uk [accessed August 2018]) (65, 66). Putative Mn binding amino acid residues in S. aureus MntE were identified based on sequence similarity to Mn binding sites previously identified in S. pneumoniae (23).

### RNA-seq analysis.

S. aureus WT strains were streaked onto TSA and incubated at 37°C for 24 h. Single colonies were selected in biological triplicate and used to inoculate 5 ml of TSB. Cultures were grown for 15 h at 37°C. Cultures were back-diluted 1:100 in 5 ml TSB and grown at 37°C until mid-log phase. Cultures were centrifuged, the supernatant was removed, and the cell pellets were resuspended in TSB. Equal volumes of the resuspended cultures were added to TSB or TSB supplemented with MnCl_2_ (final concentration of 1 mM MnCl_2_). Cells were incubated for 30 min at 37°C and centrifuged, and the remaining cell pellet was subjected to RNA extraction. RNA was isolated using Tri reagent and chloroform and precipitated with isopropanol. RNA was isolated using the Qiagen RNeasy minikit. Precipitated RNAs were treated with DNase I (Thermo Fisher) following the manufacturer’s instructions. RNA sequencing was performed by the Vanderbilt Technologies for Advanced Genomics (VANTAGE) core using the Illumina HiSeq 3000 platform (Illumina). The integrity and concentration of total RNA were determined using an Agilent 2100 Bioanalyzer system in combination with an RNA 6000 Nano kit (Agilent). rRNA was depleted using the Ribo-Zero rRNA removal kit (for bacteria) (Epicentre), and paired-end cDNA libraries were prepared with a TruSeq RNA library prep kit v2 (Illumina). Data analysis for sequencing experiments was performed on the basis of the CLC Genomics workbench (version 11.0.1; Qiagen) using the S. aureus Newman genome annotations (NCBI GenBank accession no. NC_009641.1). Prior to analysis, rRNA reads were removed in order to account for variations in rRNA depletion procedure among samples. Standard settings were used for adapter and quality trimming, as well as transcriptome sequencing (RNA-seq) analysis. Expression values were calculated as RPKM (reads per kilobase per million mapped reads) (67), and a lower cutoff of 5 RPKM was introduced for subsequent analysis.

### qRT-PCR.

Strains were streaked onto TSA and grown for 15 h at 37°C. Single colonies were selected in biological triplicate and inoculated into 5 ml of TSB and grown at 37°C for 15 h. Overnight cultures were subcultured 1:100 into TSB supplemented with 1 mM MnCl_2_ or an equal volume of sterile water and grown until mid-exponential phase. An equal volume of ice-cold acetone-ethanol was added. RNA was isolated using Tri reagent and chloroform and precipitated with isopropanol. Precipitated RNAs were treated with DNase I (Thermo Fisher) following the manufacturer’s instructions. RNA was isolated using the Qiagen RNeasy minikit. cDNA was synthesized from 2 µg RNA via incubation with Moloney murine leukemia virus (MMLV) reverse transcriptase (Thermo), using a random hexamer primer mixture. Quantitative PCR (qPCR) was performed using Bio-Rad iQ SYBR green supermix (Thermo Fisher) following the manufacturer’s instructions, using primers CMG151/152 for *gyrA*, LEJ277/278 for *mntE*, and LEJ271/2712 for *mntA*. Transcript abundance was quantified using the threshold cycle (ΔΔ*C_T_*) method after normalization to *gyrA* abundance.

### Bioluminescent reporter assay.

The bioluminescent reporter plasmid was constructed by amplifying 350 bp of the intergenic region upstream of the predicted *mntE* translational start site using primer set CMG 221/CMG 222 and cloning the fragment into the pXen-1.*luxABCDE* plasmid (p.*luxABCDE*) upstream of the *lux* operon. S. aureus WT and Δ*mntR* strains with pXen-1.*luxABCDE* or pXen-1.P*_mntE_luxABCDE* (p.P*_mntE_luxABCDE*) were streaked onto TSA-chloramphenicol and grown for 24 h at 37°C. Single colonies in biological triplicate were used to start 5-ml cultures of TSB-chloramphenicol and grown for 15 h at 37°C. One microliter of each culture was inoculated into 99 μl of TSB-chloramphenicol supplemented with 0, 125, 250, or 500 μM MnCl_2_ in 96-well clear-bottom black-walled plates and incubated at 37°C with linear shaking for 3 h. Relative bioluminescence was measured in a BioTek Cytation5 spectrophotometer with BioTek Gen5 software.

### Mn toxicity growth assays.

S. aureus strains were streaked onto TSA and grown for 24 h at 37°C. Single colonies in biological triplicate were used to start 5-ml cultures of TSB and grown at 37°C for 16 h. One microliter of each culture was added to 99 μl of TSB supplemented with 0, 0.5, or 1 mM MnCl_2_ in a 96-well flat-bottom plate. The plate was incubated at 37°C with linear shaking in a Biotek Epoch II plate reader. The optical density at 600 nm (OD_600_) of each sample was measured every hour and analyzed using BioTek Gen5 software. Using a sterile wooden applicator, liquid cultures were also streaked onto TSA or TSA supplemented with 0.5 or 1 mM MnCl_2_. Plates were incubated for 24 h at 37°C.

### ICP-MS.

S. aureus strains were streaked onto TSA and grown for 24 h at 37°C. Single colonies in biological triplicate were used to inoculate 5-ml cultures of TSB or TSB plus 100 μM EDTA. Cultures were incubated for 15 h at 37°C. Liquid cultures were back-diluted 1:100 in 5 ml TSB and incubated at 37°C for 3 h. Cells were centrifuged, the supernatant decanted, and then the cells were washed in sterile Milli-Q water (Millipore Sigma) twice. The cell pellet was resuspended in 0.5 ml Milli-Q water, and 0.5 ml of the suspension was transferred to 15-ml metal-free conical tubes. Cell suspensions were digested by the addition of 0.5 ml optimum-grade metal-free nitric acid (Thermo Fisher Scientific). Elemental quantification of acid-digested liquid samples was performed using an Agilent 7700 inductively coupled plasma mass spectrometer (Agilent, Santa Clara, CA). The following settings were fixed for the analysis: cell entrance = −40 V, cell exit = −60 V, plate bias = −60 V, OctP bias = −18 V, and collision cell helium flow = 4.5 ml/min. Optimal voltages for Extract 2, Omega Bias, Omega Lens, OctP RF, and Deflect were determined empirically before each sample set was analyzed. Element calibration curves were generated using the Aristar ICP standard mix (VWR, Radnor, PA). Samples were introduced by peristaltic pump with 0.5-mm internal diameter tubing through a MicroMist borosilicate glass nebulizer (Agilent). Samples were initially up taken at 0.5 rps for 30 s followed by 30 s at 0.1 rps to stabilize the signal. Samples were analyzed in Spectrum mode at 0.1 rps collecting three points across each peak and performing three replicates of 100 sweeps for each element analyzed. Data were acquired and analyzed using the Agilent Mass Hunter Workstation software version A.01.02.

### HOCl killing of *S. aureus*.

S. aureus stationary-phase overnight cultures were diluted 10,000-fold in biological triplicate into PBS containing 25, 12.5, 6.25, 3.125, or 0 μM NaOCl (Clorox). Samples were mixed well and incubated statically at 25°C for 30 min. Each sample was diluted 10-fold into TSB and incubated with shaking for 24 h at 37°C on a BioTek Epoch II plate reader. The optical density at 600 nm of each sample was measured every 30 min. Each sample was also serially diluted 10^0^ through 10^−5^ in 10-fold increments in PBS, and 10 μl of each dilution was spot plated on TSA. Plates were incubated for 18 h at 37°C, and CFU/ml were enumerated.

### Murine models of infections.

All animal experiments were reviewed and approved by the Vanderbilt University Institutional Animal Care and Use Committee. Procedures were performed according to the institutional policies, Animal Welfare Act, NIH guidelines, and American Veterinary Medical Association guidelines on euthanasia. S. aureus strains were streaked onto TSA and grown for 24 h at 37°C. For all experiments, 6-week-old female BALB/cJ mice were retro-orbitally infected with 1 × 10^7^ or 3 × 10^7^ S. aureus CFU in 100 μl of phosphate-buffered saline as previously described (68). Following the inoculation, the infection was allowed to proceed for up to 10 days, and then the mice were humanely euthanized. For mice infected with 1 × 10^7^ CFU, hearts, kidneys, and livers were removed 4 or 10 days postinfection to determine organ bacterial burdens. Organs were homogenized in sterile PBS using a Bullet Blender tissue homogenizer (Next Advance), and serial dilutions were plated onto TSA to enumerate CFU. To determine mortality curves, mice were infected with 3 × 10^7^ CFU, and the infection was allowed to proceed for 10 days. Natural mortalities and moribund animals that were euthanized were documented as mortality events.

### Paraquat sensitivity assays.

S. aureus strains were streaked onto TSA and incubated at 37°C for 24 h. Single colonies were selected in biological triplicate and used to inoculate 5-ml cultures of TSB. Liquid cultures were incubated for 15 h at 37°C. TSB with 0.3 and 0.6 mM paraquat was prepped using a 1 M paraquat dichloride hydrate stock prepared fresh daily with sterile Milli-Q water (Millipore Sigma). An equal volume of sterile water was added to TSB as a negative control. Two microliters of each culture was added to 198 μl of each TSB-paraquat treatment in a 96-well flat-bottom plate and incubated at 37°C with linear shaking in a BioTek Epoch II plate reader. The optical density at 600 nm of each sample was measured every 1 h and analyzed using BioTek Gen5 software. Growth curves using murine hearts and kidneys were prepared in the same manner as described above, with the exception of 2 μl of organ homogenate serving as the inoculum.

### Statistical analysis.

All data analysis and statistical tests were performed in GraphPad Prism 6 software. Replicate numbers and statistical tests for each experiment are listed in the figure captions.

### Data availability.

The data received from RNA sequencing experiments have been deposited into the NCBI Gene Expression Omnibus (accession no. GSE124285) and were consolidated into Table S2 in the supplemental material.

## References

[1] Juttukonda LJ, Skaar EP 2015 Manganese homeostasis and utilization in pathogenic bacteria. Mol Microbiol 97:216–228. doi:10.1111/mmi.13034. 25898914 PMC4631260

[2] Lisher JP, Giedroc DP 2013 Manganese acquisition and homeostasis at the host-pathogen interface. Front Cell Infect Microbiol 3:91. doi:10.3389/fcimb.2013.00091. 24367765 PMC3851752

[3] Klevens RM, Morrison MA, Nadle J, Petit S, Gershman K, Ray S, Harrison LH, Lynfield R, Dumyati G, Townes JM, Craig AS, Zell ER, Fosheim GE, McDougal LK, Carey RB, Fridkin SK, Active Bacterial Core surveillance (ABCs) MRSA Investigators. 2007 Invasive methicillin-resistant *Staphylococcus aureus* infections in the United States. JAMA 298:1763–1771. doi:10.1001/jama.298.15.1763. 17940231

[4] Hood MI, Skaar EP 2012 Nutritional immunity: transition metals at the pathogen-host interface. Nat Rev Microbiol 10:525–537. doi:10.1038/nrmicro2836. 22796883 PMC3875331

[5] Weinberg ED 1975 Nutritional immunity. Host's attempt to withhold iron from microbial invaders. JAMA 231:39–41. doi:10.1001/jama.1975.03240130021018. 1243565

[6] Damo SM, Kehl-Fie TE, Sugitani N, Holt ME, Rathi S, Murphy WJ, Zhang Y, Betz C, Hench L, Fritz G, Skaar EP, Chazin WJ 2013 Molecular basis for manganese sequestration by calprotectin and roles in the innate immune response to invading bacterial pathogens. Proc Natl Acad Sci U S A 110:3841–3846. doi:10.1073/pnas.1220341110. 23431180 PMC3593839

[7] Kehl-Fie TE, Skaar EP 2010 Nutritional immunity beyond iron: a role for manganese and zinc. Curr Opin Chem Biol 14:218–224. doi:10.1016/j.cbpa.2009.11.008. 20015678 PMC2847644

[8] Nakashige TG, Zhang B, Krebs C, Nolan EM 2015 Human calprotectin is an iron-sequestering host-defense protein. Nat Chem Biol 11:765–771. doi:10.1038/nchembio.1891. 26302479 PMC4575267

[9] Kehl-Fie TE, Chitayat S, Hood MI, Damo S, Restrepo N, Garcia C, Munro KA, Chazin WJ, Skaar EP 2011 Nutrient metal sequestration by calprotectin inhibits bacterial superoxide defense, enhancing neutrophil killing of *Staphylococcus aureus*. Cell Host Microbe 10:158–164. doi:10.1016/j.chom.2011.07.004. 21843872 PMC3157011

[10] Hunter MJ, Chazin WJ 1998 High level expression and dimer characterization of the S100 EF-hand proteins, migration inhibitory factor-related proteins 8 and 14. J Biol Chem 273:12427–12435. doi:10.1074/jbc.273.20.12427. 9575199

[11] Kehl-Fie TE, Zhang Y, Moore JL, Farrand AJ, Hood MI, Rathi S, Chazin WJ, Caprioli RM, Skaar EP 2013 MntABC and MntH contribute to systemic *Staphylococcus aureus* infection by competing with calprotectin for nutrient manganese. Infect Immun 81:3395–3405. doi:10.1128/IAI.00420-13. 23817615 PMC3754211

[12] Tipton IH, Cook MJ 1963 Trace elements in human tissue. II. Adult subjects from the United States. Health Phys 9:103–145. doi:10.1097/00004032-196302000-00002. 13985137

[13] Hurley LS, Keen CL 1987 Manganese, p 185–223. *In* Underwood E, Mertz E (ed), Trace elements in human health and animal nutrition. Academic Press, New York, NY.

[14] Juttukonda LJ, Berends ETM, Zackular JP, Moore JL, Stier MT, Zhang Y, Schmitz JE, Beavers WN, Wijers CD, Gilston BA, Kehl-Fie TE, Atkinson J, Washington MK, Peebles RS, Chazin WJ, Torres VJ, Caprioli RM, Skaar EP 2017 Dietary manganese promotes staphylococcal infection of the heart. Cell Host Microbe 22:531–542.e8. doi:10.1016/j.chom.2017.08.009. 28943329 PMC5638708

[15] Horsburgh MJ, Wharton SJ, Cox AG, Ingham E, Peacock S, Foster SJ 2002 MntR modulates expression of the PerR regulon and superoxide resistance in *Staphylococcus aureus* through control of manganese uptake. Mol Microbiol 44:1269–1286. doi:10.1046/j.1365-2958.2002.02944.x. 12028379

[16] Handke LD, Gribenko AV, Timofeyeva Y, Scully IL, Anderson AS 2018 MntC-dependent manganese transport is essential for *Staphylococcus aureus* oxidative stress resistance and virulence. mSphere 3:e00336-18. doi:10.1128/mSphere.00336-18. 30021878 PMC6052334

[17] Garcia YM, Barwinska-Sendra A, Tarrant E, Skaar EP, Waldron KJ, Kehl-Fie TE 2017 A superoxide dismutase capable of functioning with iron or manganese promotes the resistance of *Staphylococcus aureus* to calprotectin and nutritional immunity. PLoS Pathog 13:e1006125. doi:10.1371/journal.ppat.1006125. 28103306 PMC5245786

[18] Chandrangsu P, Rensing C, Helmann JD 2017 Metal homeostasis and resistance in bacteria. Nat Rev Microbiol 15:338–350. doi:10.1038/nrmicro.2017.15. 28344348 PMC5963929

[19] Turner AG, Ong CL, Gillen CM, Davies MR, West NP, McEwan AG, Walker MJ 2015 Manganese homeostasis in group A *Streptococcus* is critical for resistance to oxidative stress and virulence. mBio 6:e00278-15. doi:10.1128/mBio.00278-15. 25805729 PMC4453566

[20] Rosch JW, Gao G, Ridout G, Wang YD, Tuomanen EI 2009 Role of the manganese efflux system mntE for signalling and pathogenesis in *Streptococcus pneumoniae*. Mol Microbiol 72:12–25. doi:10.1111/j.1365-2958.2009.06638.x. 19226324 PMC2706702

[21] Xu J, Zheng C, Cao M, Zeng T, Zhao X, Shi G, Chen H, Bei W 2017 The manganese efflux system MntE contributes to the virulence of *Streptococcus suis* serotype 2. Microb Pathog 110:23–30. doi:10.1016/j.micpath.2017.06.022. 28629722

[22] Veyrier FJ, Boneca IG, Cellier MF, Taha MK 2011 A novel metal transporter mediating manganese export (MntX) regulates the Mn to Fe intracellular ratio and *Neisseria meningitidis* virulence. PLoS Pathog 7:e1002261. doi:10.1371/journal.ppat.1002261. 21980287 PMC3182930

[23] Martin JE, Giedroc DP 2016 Functional determinants of metal ion transport and selectivity in paralogous cation diffusion facilitator transporters CzcD and MntE in *Streptococcus pneumoniae*. J Bacteriol 198:1066–1076. doi:10.1128/JB.00975-15. 26787764 PMC4800876

[24] Huang X, Shin JH, Pinochet-Barros A, Su TT, Helmann JD 2017 *Bacillus subtilis* MntR coordinates the transcriptional regulation of manganese uptake and efflux systems. Mol Microbiol 103:253–268. doi:10.1111/mmi.13554. 27748968 PMC5218975

[25] Waters LS, Sandoval M, Storz G 2011 The *Escherichia coli* MntR miniregulon includes genes encoding a small protein and an efflux pump required for manganese homeostasis. J Bacteriol 193:5887–5897. doi:10.1128/JB.05872-11. 21908668 PMC3194919

[26] Li C, Tao J, Mao D, He C 2011 A novel manganese efflux system, YebN, is required for virulence by *Xanthomonas oryzae* pv. oryzae. PLoS One 6:e21983. doi:10.1371/journal.pone.0021983. 21789199 PMC3136493

[27] Wang W, Guffanti AA, Wei Y, Ito M, Krulwich TA 2000 Two types of *Bacillus subtilis tetA*(L) deletion strains reveal the physiological importance of TetA(L) in K^+^ acquisition as well as in Na^+^, alkali, and tetracycline resistance. J Bacteriol 182:2088–2095. doi:10.1128/JB.182.8.2088-2095.2000. 10735849 PMC111255

[28] Moore CM, Gaballa A, Hui M, Ye RW, Helmann JD 2005 Genetic and physiological responses of *Bacillus subtilis* to metal ion stress. Mol Microbiol 57:27–40. doi:10.1111/j.1365-2958.2005.04642.x. 15948947

[29] Grass G, Otto M, Fricke B, Haney CJ, Rensing C, Nies DH, Munkelt D 2005 FieF (YiiP) from *Escherichia coli* mediates decreased cellular accumulation of iron and relieves iron stress. Arch Microbiol 183:9–18. doi:10.1007/s00203-004-0739-4. 15549269

[30] Coady A, Xu M, Phung Q, Cheung TK, Bakalarski C, Alexander MK, Lehar SM, Kim J, Park S, Tan MW, Nishiyama M 2015 The *Staphylococcus aureus* ABC-type manganese transporter MntABC is critical for reinitiation of bacterial replication following exposure to phagocytic oxidative burst. PLoS One 10:e0138350. doi:10.1371/journal.pone.0138350. 26379037 PMC4574778

[31] Hausmann S, Guimaraes VA, Garcin D, Baumann N, Linder P, Redder P 2017 Both exo- and endo-nucleolytic activities of RNase J1 from *Staphylococcus aureus* are manganese dependent and active on triphosphorylated 5'-ends. RNA Biol 14:1431–1443. doi:10.1080/15476286.2017.1300223. 28277929 PMC5711453

[32] Radin JN, Kelliher JL, Parraga Solorzano PK, Kehl-Fie TE 2016 The two-component system ArlRS and alterations in metabolism enable *Staphylococcus aureus* to resist calprotectin-induced manganese starvation. PLoS Pathog 12:e1006040. doi:10.1371/journal.ppat.1006040. 27902777 PMC5130280

[33] Barber-Zucker S, Shaanan B, Zarivach R 2017 Transition metal binding selectivity in proteins and its correlation with the phylogenomic classification of the cation diffusion facilitator protein family. Sci Rep 7:16381. doi:10.1038/s41598-017-16777-5. 29180655 PMC5703985

[34] Aguirre JD, Culotta VC 2012 Battles with iron: manganese in oxidative stress protection. J Biol Chem 287:13541–13548. doi:10.1074/jbc.R111.312181. 22247543 PMC3340200

[35] Jiang Q, Blount BC, Ames BN 2003 5-Chlorouracil, a marker of DNA damage from hypochlorous acid during inflammation. A gas chromatography-mass spectrometry assay. J Biol Chem 278:32834–32840. doi:10.1074/jbc.M304021200. 12810714

[36] Sawyer DT, Valentine JS 1981 How super is superoxide. Acc Chem Res 14:393–400. doi:10.1021/ar00072a005.

[37] Beavers WN, Skaar EP 2016 Neutrophil-generated oxidative stress and protein damage in *Staphylococcus aureus*. Pathog Dis 74:ftw060. doi:10.1093/femspd/ftw060. 27354296 PMC5975594

[38] Vigonsky E, Fish I, Livnat-Levanon N, Ovcharenko E, Ben-Tal N, Lewinson O 2015 Metal binding spectrum and model structure of the *Bacillus anthracis* virulence determinant MntA. Metallomics 7:1407–1419. doi:10.1039/c5mt00100e. 26106847

[39] Faulkner MJ, Ma Z, Fuangthong M, Helmann JD 2012 Derepression of the *Bacillus subtilis* PerR peroxide stress response leads to iron deficiency. J Bacteriol 194:1226–1235. doi:10.1128/JB.06566-11. 22194458 PMC3294777

[40] Ma Z, Faulkner MJ, Helmann JD 2012 Origins of specificity and cross-talk in metal ion sensing by *Bacillus subtilis* Fur. Mol Microbiol 86:1144–1155. doi:10.1111/mmi.12049. 23057863 PMC3508374

[41] Chiancone E, Ceci P 2010 The multifaceted capacity of Dps proteins to combat bacterial stress conditions: detoxification of iron and hydrogen peroxide and DNA binding. Biochim Biophys Acta 1800:798–805. doi:10.1016/j.bbagen.2010.01.013. 20138126

[42] Faulkner MJ, Helmann JD 2011 Peroxide stress elicits adaptive changes in bacterial metal ion homeostasis. Antioxid Redox Signal 15:175–189. doi:10.1089/ars.2010.3682. 20977351 PMC3110094

[43] Guan G, Pinochet-Barros A, Gaballa A, Patel SJ, Argüello JM, Helmann JD 2015 PfeT, a P1B4-type ATPase, effluxes ferrous iron and protects *Bacillus subtilis* against iron intoxication. Mol Microbiol 98:787–803. doi:10.1111/mmi.13158. 26261021 PMC4648274

[44] Helmann JD 2014 Specificity of metal sensing: iron and manganese homeostasis in *Bacillus subtilis*. J Biol Chem 289:28112–28120. doi:10.1074/jbc.R114.587071. 25160631 PMC4192466

[45] Horsburgh MJ, Clements MO, Crossley H, Ingham E, Foster SJ 2001 PerR controls oxidative stress resistance and iron storage proteins and is required for virulence in *Staphylococcus aureus*. Infect Immun 69:3744–3754. doi:10.1128/IAI.69.6.3744-3754.2001. 11349039 PMC98383

[46] Greger JL 1998 Dietary standards for manganese: overlap between nutritional and toxicological studies. J Nutr 128:368S–371S. doi:10.1093/jn/128.2.368S. 9478027

[47] Stafford SL, Bokil NJ, Achard ME, Kapetanovic R, Schembri MA, McEwan AG, Sweet MJ 2013 Metal ions in macrophage antimicrobial pathways: emerging roles for zinc and copper. Biosci Rep 33:e00049. doi:10.1042/BSR20130014. 23738776 PMC3712485

[48] Djoko KY, Ong CL, Walker MJ, McEwan AG 2015 The role of copper and zinc toxicity in innate immune defense against bacterial pathogens. J Biol Chem 290:18954–18961. doi:10.1074/jbc.R115.647099. 26055706 PMC4521016

[49] Forbes JR, Gros P 2003 Iron, manganese, and cobalt transport by Nramp1 (Slc11a1) and Nramp2 (Slc11a2) expressed at the plasma membrane. Blood 102:1884–1892. doi:10.1182/blood-2003-02-0425. 12750164

[50] Jabado N, Jankowski A, Dougaparsad S, Picard V, Grinstein S, Gros P 2000 Natural resistance to intracellular infections: natural resistance-associated macrophage protein 1 (Nramp1) functions as a pH-dependent manganese transporter at the phagosomal membrane. J Exp Med 192:1237–1248. doi:10.1084/jem.192.9.1237. 11067873 PMC2193348

[51] Nairz M, Haschka D, Demetz E, Weiss G 2014 Iron at the interface of immunity and infection. Front Pharmacol 5:152. doi:10.3389/fphar.2014.00152. 25076907 PMC4100575

[52] Painter KL, Strange E, Parkhill J, Bamford KB, Armstrong-James D, Edwards AM 2015 *Staphylococcus aureus* adapts to oxidative stress by producing H_2_O_2_-resistant small-colony variants via the SOS response. Infect Immun 83:1830–1844. doi:10.1128/IAI.03016-14. 25690100 PMC4399076

[53] Johnson M, Sengupta M, Purves J, Tarrant E, Williams PH, Cockayne A, Muthaiyan A, Stephenson R, Ledala N, Wilkinson BJ, Jayaswal RK, Morrissey JA 2011 Fur is required for the activation of virulence gene expression through the induction of the *sae* regulatory system in *Staphylococcus aureus*. Int J Med Microbiol 301:44–52. doi:10.1016/j.ijmm.2010.05.003. 20705504 PMC2994983

[54] Geiger T, Goerke C, Mainiero M, Kraus D, Wolz C 2008 The virulence regulator Sae of *Staphylococcus aureus*: promoter activities and response to phagocytosis-related signals. J Bacteriol 190:3419–3428. doi:10.1128/JB.01927-07. 18344360 PMC2395011

[55] Voyich JM, Vuong C, DeWald M, Nygaard TK, Kocianova S, Griffith S, Jones J, Iverson C, Sturdevant DE, Braughton KR, Whitney AR, Otto M, DeLeo FR 2009 The SaeR/S gene regulatory system is essential for innate immune evasion by *Staphylococcus aureus*. J Infect Dis 199:1698–1706. doi:10.1086/598967. 19374556 PMC2799113

[56] Voyich JM, Braughton KR, Sturdevant DE, Whitney AR, Said-Salim B, Porcella SF, Long RD, Dorward DW, Gardner DJ, Kreiswirth BN, Musser JM, DeLeo FR 2005 Insights into mechanisms used by *Staphylococcus aureus* to avoid destruction by human neutrophils. J Immunol 175:3907–3919. doi:10.4049/jimmunol.175.6.3907. 16148137

[57] Traber KE, Lee E, Benson S, Corrigan R, Cantera M, Shopsin B, Novick RP 2008 agr function in clinical *Staphylococcus aureus* isolates. Microbiology 154:2265–2274. doi:10.1099/mic.0.2007/011874-0. 18667559 PMC4904715

[58] Torres VJ, Attia AS, Mason WJ, Hood MI, Corbin BD, Beasley FC, Anderson KL, Stauff DL, McDonald WH, Zimmerman LJ, Friedman DB, Heinrichs DE, Dunman PM, Skaar EP 2010 *Staphylococcus aureus fur* regulates the expression of virulence factors that contribute to the pathogenesis of pneumonia. Infect Immun 78:1618–1628. doi:10.1128/IAI.01423-09. 20100857 PMC2849423

[59] Choby JE, Mike LA, Mashruwala AA, Dutter BF, Dunman PM, Sulikowski GA, Boyd JM, Skaar EP 2016 A small-molecule inhibitor of iron-sulfur cluster assembly uncovers a link between virulence regulation and metabolism in *Staphylococcus aureus*. Cell Chem Biol 23:1351–1361. doi:10.1016/j.chembiol.2016.09.012. 27773628 PMC5117899

[60] Fey PD, Endres JL, Yajjala VK, Widhelm TJ, Boissy RJ, Bose JL, Bayles KW 2013 A genetic resource for rapid and comprehensive phenotype screening of nonessential *Staphylococcus aureus* genes. mBio 4:e00537-12. doi:10.1128/mBio.00537-12. 23404398 PMC3573662

[61] Overbeek R, Olson R, Pusch GD, Olsen GJ, Davis JJ, Disz T, Edwards RA, Gerdes S, Parrello B, Shukla M, Vonstein V, Wattam AR, Xia F, Stevens R 2014 The SEED and the Rapid Annotation of microbial genomes using Subsystems Technology (RAST). Nucleic Acids Res 42:D206–D214. doi:10.1093/nar/gkt1226. 24293654 PMC3965101

[62] Sievers F, Wilm A, Dineen D, Gibson TJ, Karplus K, Li W, Lopez R, McWilliam H, Remmert M, Soding J, Thompson JD, Higgins DG 2011 Fast, scalable generation of high-quality protein multiple sequence alignments using Clustal Omega. Mol Syst Biol 7:539. doi:10.1038/msb.2011.75. 21988835 PMC3261699

[63] Kumar S, Stecher G, Tamura K 2016 MEGA7: Molecular Evolutionary Genetics Analysis version 7.0 for bigger datasets. Mol Biol Evol 33:1870–1874. doi:10.1093/molbev/msw054. 27004904 PMC8210823

[64] Kelley LA, Mezulis S, Yates CM, Wass MN, Sternberg MJ 2015 The Phyre2 web portal for protein modeling, prediction and analysis. Nat Protoc 10:845–858. doi:10.1038/nprot.2015.053. 25950237 PMC5298202

[65] Jones DT 1999 Protein secondary structure prediction based on position-specific scoring matrices. J Mol Biol 292:195–202. doi:10.1006/jmbi.1999.3091. 10493868

[66] Nugent T, Jones DT 2009 Transmembrane protein topology prediction using support vector machines. BMC Bioinformatics 10:159. doi:10.1186/1471-2105-10-159. 19470175 PMC2700806

[67] Mortazavi A, Williams BA, McCue K, Schaeffer L, Wold B 2008 Mapping and quantifying mammalian transcriptomes by RNA-Seq. Nat Methods 5:621–628. doi:10.1038/nmeth.1226. 18516045 PMC13303166

[68] Corbin BD, Seeley EH, Raab A, Feldmann J, Miller MR, Torres VJ, Anderson KL, Dattilo BM, Dunman PM, Gerads R, Caprioli RM, Nacken W, Chazin WJ, Skaar EP 2008 Metal chelation and inhibition of bacterial growth in tissue abscesses. Science 319:962–965. doi:10.1126/science.1152449. 18276893

[69] Duthie ES, Lorenz LL 1952 Staphylococcal coagulase; mode of action and antigenicity. J Gen Microbiol 6:95–107. doi:10.1099/00221287-6-1-2-95. 14927856

[70] Kreiswirth BN, Lofdahl S, Betley MJ, O'Reilly M, Schlievert PM, Bergdoll MS, Novick RP 1983 The toxic shock syndrome exotoxin structural gene is not detectably transmitted by a prophage. Nature 305:709–712. doi:10.1038/305709a0. 6226876

