# Supplementary material for: Manganese Detoxification by MntE Is Critical for Resistance to Oxidative Stress and Virulence of Staphylococcus aureus
Source: mBio. 2019 Feb 26;10(1):e02915-18. doi: 10.1128/mBio.02915-18 (PMC6391924; doi:10.1128/mBio.02915-18)
Supplement: FIG S1 [file mBio.02915-18-sf001.pdf]

```
                              % Identity
Staphylococcus aureus               --MSHNENLKLAQRGAYLSLIVYIILSIVKYVTGFVFNSAAVRADALNNMTDIIVSLAVI   58
Streptococcus pyogenes        (41%) MTQDPIANLKLARKGPIVSIIVYLSLSVAKLLAGYLLNASSLIADGFNNLSDIVGNVALL   60
Streptococcus pneumoniae      (80%) -MKQSISNLKLAERGAIISISTYLILSAAKLAAGHLLHSSSLVADGFNNVSDIIGNVALL   59
Streptococcus suis            (45%) -MNQNVTNLKLAERGALLSIGAYIVLSGIKLVAGQLFHSDALRADAFNNISDIIGNIAVL   59
                                           *****  *   *   *  **  *   *     **  **  **  **    *

Staphylococcus aureus               IGLKISIKPADRNHPYGHLKSENISSLLVSFVIMFVGIQVVIQNAPRLFKEDDVVPNAIT   118
Streptococcus pyogenes              IGLHLASQPADANHKFGHWKIEDLSSLVTSFIMFLVGFQVLIHTIKSIFSGQQVDIDPLG   120
Streptococcus pneumoniae            IGIRMARQPADRDHRFGHWKIEDLASLITSIIMFYVGFDVLRDTIQKILSREETVIDPLG   119
Streptococcus suis                  VGLKMAQKPADTDHKFGHWKMEDLASLITSFIMFVVGFQVLYDTLQKLISNSSIEVDIMG   119
                                     *      ***  *  ** * *   **  *     **  *

Staphylococcus aureus               IIVSLISGLVMLIVFAVNQRLAKRTKSSSLNSAAKDNLSDSLVSIGTAIGLIFTQIGFPI   178
Streptococcus pyogenes              AIVGIVSAFVMLGVYVFNKRLSKRVKSSALVAASKDNLADAVTSIGTSIAIIAASLHLPV   180
Streptococcus pneumoniae            ATLGIISAAIMFVVYLYNTRLSKKSNSNALKAAAKDNLSDAVTSLGTAIAILASSFNYPI   179
Streptococcus suis                  AIVGIFSALVMLAVYLYNNRLAKKVRSKALEAAAKDNLSDAVTSIGTSIAIFAAAFNFPI   179
                                          *   *  *   * ** *   *  *  * **** *   * ** *         *

Staphylococcus aureus               VDIILATLLGLLIVYTGFGIFKEAIFMLSDGFNETELEAYRNDILEVDEVQEVKSIKGRY   238
Streptococcus pyogenes              IDHIAAMIITFFILKTAFDIFMESSFSLSDGFDSRHLKKYEKAILEIPKIVAVKSQRART   240
Streptococcus pneumoniae            VDKLVAIIITFFILKTAYDIFIESSFSLSDGFDDRLLEDYQKAIMEIPKISKVKSQRGRT   239
Streptococcus suis                  VDKIAAIIITFFILKTAYDIFMESFFTLSDGFDENLLKKYEEDILKLPKIVSVKSQRGRT   239
                                     *   *      *  *   ** *  * *****    *  *   *        ***   *

Staphylococcus aureus               HGSSVFIDVTIVVDANLSLVEAHQICDNVEHHLHK-KGISSVYVHPEPDHL---------   288
Streptococcus pyogenes              YGSNVYLDIVLEMNPDLSVYESHSITEKVEQLLSDQFSIYDIDIHVEPAMIPEEEIFDNV   300
Streptococcus pneumoniae            YGSNIYLDITLEMNPDLSVFESHEIADQVESMLEERFGVFDTDVHIEPAPIPEDEILDNV   299
Streptococcus suis                  YGANIYLDVVLEMNPDLSVYESHEVTEQVEQLLTLKHGVFDVDIHVEPSEIPHDEMYEHV   299
                                     *     *        **  * *     **  *           * **

Staphylococcus aureus               ------------------------------------------------------------   288
Streptococcus pyogenes              AKKLYRYEKLILSKVPDYDHYIAKSFQLIDANGQTVNYEQFLNQEIYYPSNFNHFQIESI   360
Streptococcus pneumoniae            YKKLLMREQL-IDQGNQLEELLTDDFVYIRQDGEQMDKEAYKTKKE-LNSAIKDIQITSI   357
Streptococcus suis                  YDKLFRFETEIQAHANGYEELIDDQYLLIDAKGRYRNKTQMLADHPIQTTYLSNYQMTSI   359

Staphylococcus aureus               -----------------------------------------------------   288
Streptococcus pyogenes              SQKTMLVTYQLNGNQRTSIWRRHESWSLLFHQITPIAKKQLHHTHYRIVKM--   411
Streptococcus pneumoniae            SQKTKLICYELDGIIHTSIWRRHETWQNIFHQETKKE----------------   394
Streptococcus suis                  SQKSKLVTFEIGDYVHTSLWRRHENWTVIFHQISKKQASQIEATEKSSDLHNP   412
```
