# Supplementary figures and images for: Manganese Detoxification by MntE Is Critical for Resistance to Oxidative Stress and Virulence of Staphylococcus aureus
Source: mBio. 2019 Feb 26;10(1):e02915-18. doi: 10.1128/mBio.02915-18 (PMC6391924; doi:10.1128/mBio.02915-18)

**A**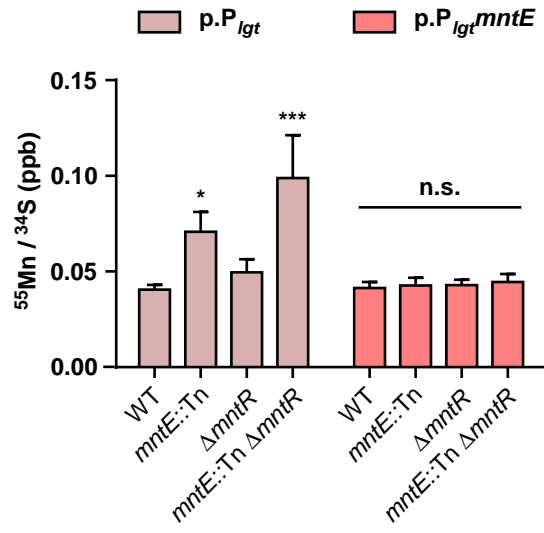**B**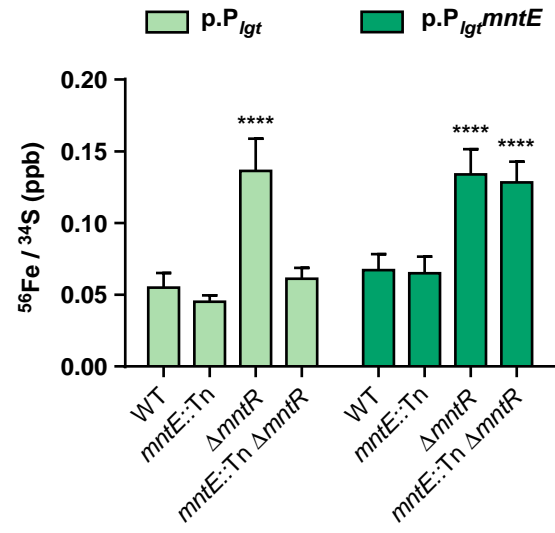

Supplement: FIG S2 [file mBio.02915-18-sf002.pdf]

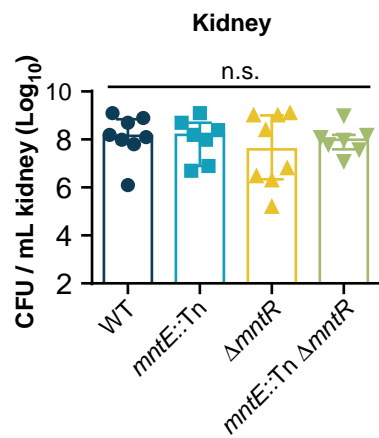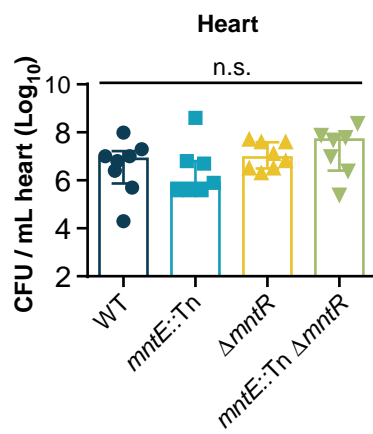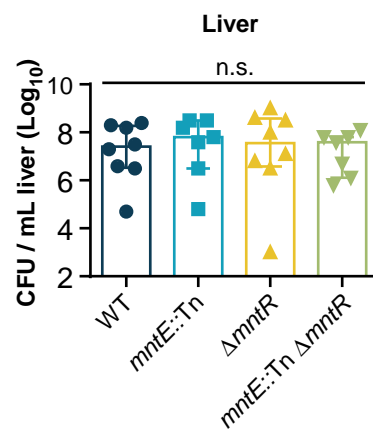

Supplement: FIG S3 [file mBio.02915-18-sf003.pdf]

**A**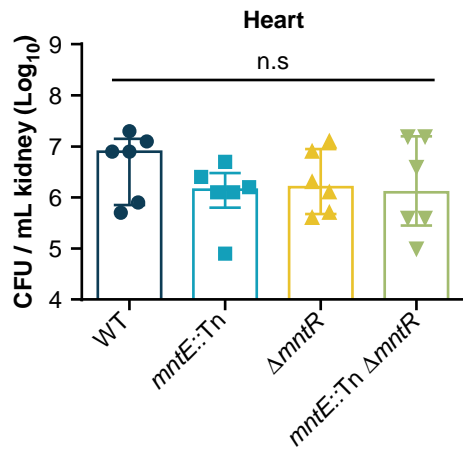**B**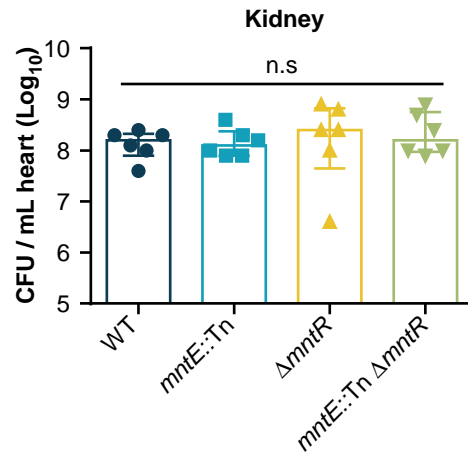

Supplement: FIG S5 [file mBio.02915-18-sf005.pdf]
