# Supplementary material for: Manganese Detoxification by MntE Is Critical for Resistance to Oxidative Stress and Virulence of Staphylococcus aureus
Source: mBio. 2019 Feb 26;10(1):e02915-18. doi: 10.1128/mBio.02915-18 (PMC6391924; doi:10.1128/mBio.02915-18)
Supplement: FIG S4 [file mBio.02915-18-sf004.pdf]

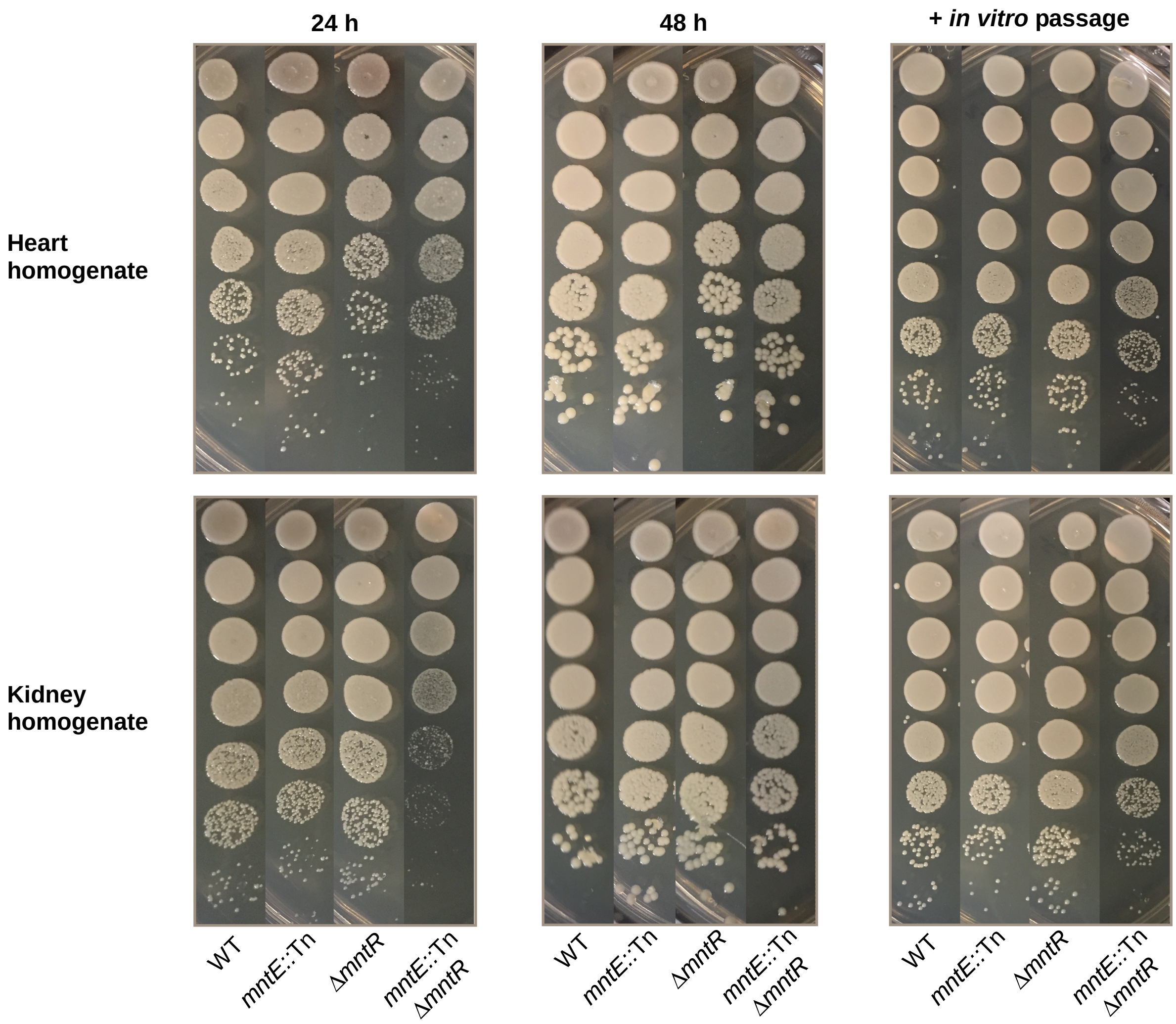

24 h
48 h
+ *in vitro* passage
Heart homogenate
Kidney homogenate
WT
mntE::Tn
Δ*mntR*
mntE::Tn Δ*mntR*
WT
mntE::Tn
Δ*mntR*
mntE::Tn Δ*mntR*
WT
mntE::Tn
Δ*mntR*
mntE::Tn Δ*mntR*
