# Supplementary material for: Manganese Detoxification by MntE Is Critical for Resistance to Oxidative Stress and Virulence of Staphylococcus aureus
Source: mBio. 2019 Feb 26;10(1):e02915-18. doi: 10.1128/mBio.02915-18 (PMC6391924; doi:10.1128/mBio.02915-18)
Supplement: TABLE S2 [file mBio.02915-18-st002.docx]

**Supplemental Table 2. Plasmids and primers used in this study.**

| **Plasmids** | | | |
| --- | --- | --- | --- |
| **Plasmid** | | **Description** | **Source** |
| pXen1 (*luxABCDE*) | | Promoterless plasmid encoding *Photorhabdus luminescens* luciferase operon (*luxABCDE*), carbenicillin (*E. coli*) and chloramphenicol (*S. aureus*) selection | Xenogen; (6) |
| pXen1 P*_mntE_luxABCDE* | | 350 bp upstream of *mntE* start codon cloned in pXen1 | This study |
| pOS1 P*_lgt_* | | *lgt* (constitutive) promoter | (8) |
| pOS1 P*_lgt_mntE* | | *mntE* cloned in pOS1 P*_lgt_* | This study |
| **Primers** | | | |
| **Primer name** | **Sequence** | | |
| CMG_163 | aaatacaattgaggtgaacatATGTCTCATAATGAAAATCTTAAATTG | | |
| CMG_164 | aaacactacccccttgtttggatccTTATAGATGGTCTGGTTCTGG | | |
| CMG_176 | GTTGCTGCCCCAACTAGCAAC | | |
| CMG_177 | CCACTCGCTTTGTCCATGACG | | |
| LEJ_277 | ctgcagctgtaagagccgatg | | |
| LEJ_278 | aggatgatttcgatcggcagg | | |
| LEJ_271 | tctagatgagccgtttgtcg | | |
| LEJ_272 | gcttttgatagatcatggtgga | | |
| CMG_221 | agtcctcctcttgcttcatctgcaggatccgcgttgaaccaccttgaattag | | |
| CMG_222 | cgacgttgtaaaacgacggccagtgaattcACAAATAAATTTGAAGAAGACGG | | |
